# Helminth reshapes host gut microbiota and immunoregulation by deploying an antimicrobial program of innate immunity

**DOI:** 10.1080/19490976.2025.2496447

**Published:** 2025-04-23

**Authors:** Yang Zou, Lixia Pu, Aijiang Guo, Yaqi Li, Yihui Liu, Yugui Wang, Yingying Ding, Xiaowei Du, Xiaola Guo, Shaohua Zhang, Xuepeng Cai, Shuai Wang

**Affiliations:** a State Key Laboratory of Animal Disease Control and Prevention, College of Veterinary Medicine, Lanzhou University, Lanzhou Veterinary Research Institute, Chinese Academy of Agricultural Sciences, Lanzhou, Gansu, China; b Key Laboratory of Veterinary Parasitology of Gansu Province, Lanzhou Veterinary Research Institute, Chinese Academy of Agricultural Sciences, Lanzhou, Gansu, China

**Keywords:** Helminths, gut microbiota, macrophages, immunoregulation

## Abstract

Helminths can manipulate their host’s gut microbiota, with the expansion of the lactobacilli population being a common feature. This process profoundly influences host immunoregulation, yet the underlying mechanisms remain almost unknown. Using a tissue-dwelling helminth model (larval *Echinococcus multilocularis*) while validating key findings from other helminth infections, we show that helminths harness the antibacterial program of host innate immunity to transform the host gut microbiome and control gut microbiota-mediated immunity. Using multifaceted techniques, we elucidate that cathelicidin-related antimicrobial peptide (CRAMP), derived from the expanded CD11b^+^CD206^+^ macrophages rather than the intestinal epithelial cells, is the key component that enters into the gut ecological system and enhances the fitness of *Lactobacillus* by selectively killing gram-negative microbes like enterobacteria. Furthermore, through *in vitro* cell culturing and *in vivo* dietary intervention experiments, we demonstrate that this regulation from innate immunity is boosted via toll-like receptor signaling by helminth’s secretory products, which could be sufficiently tuned down by dietary vitamin D through its receptor and cyp27b1. Importantly, using microbiota-targeted treatment methods, we prove that this signaling bolsters gut microbiota-mediated host intestinal Foxp3^+^ Treg cell expansion and parasite survival and that therapies targeting this signaling are effective in treating infection. We outline a dietary micronutrient-dependent mechanism by which helminths leverage host innate immunity to edit the host gut microbiome and thereby control immunosuppression precisely.

## Introduction

Helminths are among the most important infectious agents, globally affecting at least 2 billion people.^1^ Inhabiting gastrointestinal tracts (GI) or dwelling in tissues, they have co-evolved with their hosts and symbiotic microbiota (e.g., bacteria).^2^ During helminth infection, the host gut microbiome is markedly altered structurally and functionally under experimental and natural conditions.^2^ The interactions between the symbiotes likely profoundly affect host health and disease. The remarkable suppressive properties of helminths on parasite survival as well as autoimmunity and allergic diseases^3,4^ have been suggested to stem, at least partially, from direct and/or indirect cross-talk between the parasites and the host’s microbes,^5–7^ particularly by promoting central immune cells like regulatory T cells (Treg).^8^ This hypothesis is supported by the discovery of causal relationships in various helminth infection models, such as murine intestinal nematodes, in which the infections change the host microbiota to shape host immunity.^9–13^ However, the exact mechanisms of how helminth infections remodel the intestinal microbial communities of the host are still poorly understood.

The host, life cycle, size, and niche that vary drastically between helminth species and of course will impact their interaction with the microbiota population of the host. However, certain findings have been repeatedly reported in different luminal- and tissue-dwelling helminth species. Among these, the expansion of lactobacilli populations and a concomitant increase of these microbe-related metabolites-short-chain fatty acids (SCFAs, which are strong inducers of Treg)^2,5^ are the most frequently reported observations after helminth colonization (as reviewed in^5^ with species such as *Nippostrongylus brasiliensis*, *Heligmosomoides polygyrus*, *Trichuris muris*, *Ascaris suum*, and *Schistosoma mansoni*. Certain species of the genus *Lactobacillus* can exert immunoregulatory effects in many autoimmune-related diseases and promote the persistence of helminth infection (e.g., *H. polygyrus)* through enhancing Treg differentiation.^14–16^ These studies highlight that the remodeling of the gut microbiota by helminths is indicated by the expansion of lactobacilli commensals, implying a potential common mechanism underlying gut microbiota dynamics during helminth infections. Thus, dissecting the relevant mechanisms underlying lactobacilli expansion is crucial for understanding the impact of helminths on the host gut microbiota and immunoregulation.

In this study, we employed a cestode helminth model tissue-dwelling larval *Echinococcus multilocularis* in mice and validations of key findings in other helminth infection systems to dissect the mechanism underlying the host gut microbiota reshaping during infection. Being distinct from luminal-dwelling helminths, this peritoneal infection model is ideal for exploring mechanisms free from direct physical interconnections and perturbations from the parasites’ bodies or excretory/secretory products (ESP). Our study dissects an interesting relationship that the innate immune system, which is supposed to protect the host from helminth infection, is likely evolutionarily adopted by the helminths to boost suppressive immunity by selectively influencing certain symbiotic microorganisms of the host.

## Materials and methods

### Mice and infection model

The specific-pathogen-free (SPF) C57BL/6 mice (6–7 weeks old) were purchased from Vital River Laboratory Animal Technology Co., Ltd. (Beijing, China) and housed in the animal facility of Laboratory Animal Center of Lanzhou Veterinary Research Institute for at least 2 weeks before any experiments. The *cramp*-knockout (*Cnlp*^−/−^) and wild-type (WT) C57BL/6 mice were purchased from Saiye Biotechnology Co., Ltd. (Jiangsu, China) and bred in our facility. Heterozygous mice were crossed in-house to generate the *Cnlp*^−/−^ mice and age- and sex-matched WT littermates (7–9 weeks old) served as controls. Genotypes of all the homozygotes were identified by PCR following the recommended protocols. All the mice were maintained under pathogen-free conditions (under-controlled conditions of 22  ±  2°C, 40  ±  10% humidity, and a 12-h light/12-h dark cycle). All animal studies, including the euthanasia procedure, followed the regulations and guidelines of the Institutional Animal Care and Use Committee of Lanzhou Veterinary Research Institute (NO.LVRIAEC-2020-009).

The ‘Qinghai’ isolate of *E. multilocularis* (*Emu*)^17^ was used in this study. The protoscoleces of *Emu* (metacestode stage) were collected from hydatid cysts obtained from Mongolian gerbils (*Meriones unguiculatus*) that had been intraperitoneally inoculated with larval *Emu* for 12 weeks, following the previously reported method.^18^ For the infection model, mice were intraperitoneally injected with 2,000 *Emu* protoscoleces in 200 μL of phosphate-buffered saline (PBS) or with the same volume of PBS (vehicle control). The cysts typically reside in the peritoneal cavity of the infected mice. The mice were euthanized and collected for samples at 6 weeks or 12 weeks post-infection for corresponding experiments.

### Genomic DNA and mRNA extraction

For collecting gut content, the colon tissues were excised from the euthanized mice, opened longitudinally, and the colon gut content was collected. All collected samples, including mouse gut content, fecal samples, and bacterial cultures were stored at  −80°C before use. Cells and tissues were stored in liquid nitrogen. Genomic DNA was extracted using the QIAamp PowerFecal Pro Kit (Cat. 47016, Qiagen) as per the manufacturer’s protocol. The total mRNA for tissues was extracted using TRIzol Reagent (Cat. 16018, Invitrogen) according to the manufacturer’s protocol. DNA or RNA concentration was determined using the Qubit 4.0 Fluorometer (ThermoFisher Scientific).

### Isolation and culture of bacteria strains

To isolate the *Lactobacillus* and *Escherichia* species in the mice, feces from naive and infected mice were collected and homogenized in sterile PBS (100 mg in 1 mL). The homogenates were serially diluted and plated onto MRS or LB agar plates, respectively, followed by incubation with an anaerobic chamber at 37°C for 24 h. Colonies on agar plates were picked and cultured anaerobically on a medium at 37°C for 24 h. Genomic DNA was extracted from the isolates and amplified by PCR using *Lactobacillus* or Enterobacteriaceae-specific primers (Table S1), as previously described.^19^ The sequences of PCR products were determined by Sanger sequencing (TSINCKE) and aligned using BLASTn against the NCBI ‘nr’ database to identify species. The isolates were stored and the frequencies of each main species were counted.

For the *in vivo* experiments involving bacteria gavaging, bacteria were cultured in a corresponding medium and harvested at the exponential phase. For the experiment to access the immunoregulatory role of *Limosilactobacillus reuteri*, each mouse was treated with antibiotics cocktails for 5 days (see FMT part) and then orally gavaged with 2 × 10^9^ CFUs of *L. reuteri* isolate in 200 μL PBS or vehicle control (PBS) daily for 2 weeks. Four weeks after *L. reuteri* gavage, the mice were euthanized.

### Quantitative real-time PCR (qPCR) analysis of bacteria

Levels of *Lactobacillus*, Enterobacteriaceae, *Escherichia coli*, and *L. reuteri* in the mice were quantified using qPCR with primers shown in Table S1. The qPCR was performed with the following settings: 100 ng of DNA, 10 μL of SYBR Green mix, 0.4 μL of each primer (10 μM), and 7.2 μL of RNA-free water, making a total volume of 20 μL. The qPCR was carried out starting with a 95°C enzyme activation step for 2 min, followed by 40 cycles of a two-step cycling protocol (95°C for 15 s and 60°C for 1 min), using the GoTaq® qPCR Master Mix Kit (Cat. A6002, Promega) on an ABI7500 machine (ABI, USA). A standard curve of Ct value versus arbitrary units was generated by running a serially diluted pool of DNA (1:1) extracted from *E. coli* and *L. reuteri* culture alongside the analysis.

### Measurement of antibacterial peptides (AMPs) using ELISA

For colon tissues, the colon was excised from mice and opened longitudinally. The gut contents as well as adipose tissues were meticulously removed. The colon was equally divided into three parts according to the distance to the cecum (proximal, middle, and distal). The middle portion of the colon tissues and feces was weighed and homogenized in PBS (100 mg per 1 mL) and centrifuged at 3,000 rpm and 4°C for 15 min and the supernatants were collected. For blood samples, serum was collected from submandibular blood. For fecal samples, fresh fecal pellets were collected, weighed, homogenized in PBS (100 mg/mL), and centrifuged at 5,000 rpm and 4°C for 10 min, followed by collecting the supernatant. The level of each antibacterial peptide was evaluated using ELISA methods as per the recommendation of each manufacturer, including CRAMP ELISA kit (Cat. CSB-E5061m, Cusabio), Reg3g ELISA kit (Cat. CSB-EL9549MO, Cusabio), Sprr2a ELISA kit (Cat. K8–14835, KIRbio), Lyz1 ELISA kit (Cat. K8–14839), Retnla ELISA kit (Cat. K8–02975), α-defensins ELISA kit (Cat. K6–02404, KIRbio), β-defensins ELISA kit (Cat. K6–02440, KIRbio), Reg3α ELISA kit (Cat. K6–14918, KIRbio), and human LL-37 ELISA kit (Cat. CSB-EL4476HU, Cusabio).

### The *in* *vitro* and *in* *vivo* CRAMP treatment experiments

To examine whether the circulating CRAMP could affect the gut microbiome, the mice were intraperitoneally injected with murine CRAMP (mCRAMP) (5 mg/kg body weight) in 200 μL PBS or vehicle control (PBS). The feces were collected 2 h after the injection and were accessed for mCRAMP level in feces using ELISA.

The bacteria culture at logarithmic phage (OD600 = 0.6) was collected and centrifuged at 3,000 rpm for 10 min at 4°C. For the *in vitro* experiments to test the inhibition rates of mCRAMP, each of the bacteria isolates (5  ×  10^3^ CFU) was cultured in the medium with different concentrations of mCRAMP dissolved in PBS (0 μg/mL, 40 μg/mL, and 100 μg/mL) for 2 h at 37°C, respectively. The culture was then plated on agar plates for 24 h and counted for colonies. For *in vivo* experiments, an injection of a dose of 5 mg/kg for each mouse every 4 days for 4 times was used. In the *in vivo* competition experiment of *L. reuteri* and *E. coli* isolates, the mice were gavaged with the 200 μL antibiotic cocktail once (see FMT part), and 1 day later (day 0), the mice were gavaged with a mixture of 1  ×  10^8^ CFUs of each bacterial isolate (1:1 ratio). From day 0, the mice were treated with mCRAMP by IP injection every 4 days for 4 times, and the mice were euthanized at day 14 when the trend for comparison was stable.

### Bacteriophage isolation and amplification

A phage strain against *E. coli* (named EC_RND4001) was isolated from fecal samples of *Rattus norvegicus domestica*. Briefly, the fecal pellets were homogenized in SM buffer (200 mm NaCl, 16 mm MgSO4, and 0.1 M Tris-HCl, pH = 7.4) and centrifuged at 5,000 rpm for 10 min at 4°C to remove any remaining solids. The supernatant was then sequentially filtered through a 0.22 μm PVDF filter. The processed samples were cocultured with the *E. coli* isolate for 24 h at 37°C by the double-layer plate (0.4% agar and 1.5% agar) method to isolate page colonies. The resulting plaques were recovered using a sterile pipette tip in 500 μL SM buffer and then cocultured with *E. coli* at logarithmic phase (OD600 = 0.6) in 5 mL LB medium for enrichment. After overnight growth at 37°C, the lysates were centrifuged at 12,000 rpm for 10 min at 4°C. The supernatant was filtered through a 0.22 μm membrane filter. High-titer phage stocks were generated by adding 1 mL of the above phage to 50 mL *E. coli* culture. The filtered supernatants were enriched into 4 mL using a centrifugal filter (100 KD, Cat. UFC0096, Merck-Millipore) and then measured for the phage titer. For the phage experiments *in vivo*, the mice were gavaged with the phage strain ‘EC_RND4001’ or the control (autoclaved phage) every 3 days until euthanasia. Mice were gavaged with 100 μL NaHCO_3_ (1 M, pH = 8.5) to neutralize gastric acid, followed by 1  ×  10^9^ pfu of the phage in 200 μL PBS. Fecal samples were collected periodically throughout the experiment, from which bacteria were quantified using molecular methods.

### Isolation of peripheral blood cells

Fresh submandibular blood was collected from the mice. Peripheral blood mononuclear cells (PBMCs) and neutrophils in peripheral blood were separated and harvested using a mouse peripheral blood cell isolation kit (Cat. LZS1100, TBD).

### BMDM cell isolation

Murine bone marrow-derived macrophages (BMDMs) were isolated and purified using the method that has been previously described in ref 20. In brief, the euthanized mice were thoroughly disinfected with 70% ethanol, followed by the isolation of the femur and tibia from the mouse. The bone marrow was flushed into a 50 mL tube using Dulbecco’s Modified Eagle Medium (DMEM) (Cat.15092, Gibco) supplemented with 100 U/mL penicillin and 0.1 mg/mL streptomycin (1% PS), followed by filtering through a 70 μm cell strainer to separate any remaining clumps. The cells were then centrifuged at 1,500 rpm and 4°C for 10 min and resuspended in 2 mL of red blood cell lysis buffer to eliminate red blood cells. The cells were cultured with DMEM medium with 1% PBS and 10% fetal bovine serum (FBS, ultra-low endotoxin) (Cat. Z7180FBS–100, ZETA) in a 37°C, 5% CO₂ incubator for 4 h in TC-treated dishes. The non-adherent cells were collected from the medium and were centrifuged at 1,500 rpm and 4°C for 10 min. Approximately, 6  ×  10^6^ cells per petri dish in 10 mL of DMEM/F12–10 (containing 20% L929 conditioned medium, 10% FBS, and 1% PS) were seeded in T75 culture flasks (TC non-treated) and incubated at 37°C in a 5% CO₂ incubator for 7 days. On the fourth day, an additional 5 mL of DMEM/F12–10 (containing 20% L929 conditioned medium, 10% FBS, and 1% PS) was added to the culture flask. For dissociation, a gentle cell dissociation reagent (Cat. 07174, Stemcell) was pre-warmed to 37°C and added to each culture flask at a volume of 5 mL for 5 min.

### Excretory/secretory product (ESP) collection

Cysts were isolated from the peritoneal cavity of the mice that had been infected with *Emu* for 3 months. Excess surface tissue was meticulously removed and blood was washed away. The cyst tissues were then resuspended in 20 mL of DMEM medium supplemented with 100 U/mL penicillin and 0.1 mg/mL streptomycin and cultured at 37°C, 5% CO₂ incubator. The medium was collected every 8 h and 1% protease inhibitors were included in each collection. The collected excretory-secretory products were concentrated using a 3 kDa ultrafiltration tube at 4°C and 4,000 rpm for 10 min. After concentration, the ESP was washed three times with ultra-low endotoxin PBS (Cat. C3581–0500, VivaCell) to minimize phenol red contamination. The endotoxin levels in the washed ESP were measured using ToxinSensorTM Chromogenic LAL Endotoxin Assay Kit (Cat. L0350C, Genscript). Subsequently, the endotoxin content was reduced to below 0.1 Endotoxin Units (EU)/mL by Pierce™ High Capacity Endotoxin Removal Resin kit (Cat. 88270, Thermo Scientific). The protein concentration was determined by Pierce^@^ BCA protein Assay kit (Cat. 23227, ThermoFisher Scientific).

### ESP treatment on BMDMs

BMDM cells were counted and assessed for viability by Trypan Blue staining using an automated cell counter (Bio-Rad). One mL of 1  ×  10^6^ BMDMs was seeded per well in 12-well plates and incubated in fresh medium (DMEM/1% PS/10% FBS) for 3 h at 37°C in a 5% CO₂ incubator to facilitate adherence. After 3 h, the culture medium was removed, and the plates were washed with low-endotoxin PBS. BMDMs were then stimulated with 25-(OH)D_3_ (500 nM) (Cat. H4014-1 MG, Sigma-Aldrich), 1.25-(OH)_2_D_3_ (1 nM) (Cat. D1530-10UG, Sigma-Aldrich), ZK9222 (1 μM) (Cat. HY-12397, MCE), and ESP (5 μg/mL), or an equivalent volume of solvent control, for 24 h in fresh medium (DMEM/1% PS/10% FBS) at 37°C in a 5% CO₂ incubator. Following stimulation, the culture medium was collected by centrifugation at 5,000 rpm at 4°C for 15 min. The BMDMs were harvested, resuspended in 1 mL of TRIzol Reagent, and stored at  −80°C for subsequent qRT-PCR analysis. For additional *in vitro* experiments for *Tlr2* inhibition, 5  ×  10^5^ BMDMs were seeded per well in 12-well plates and stimulated with C29 (100 μM) (Cat. inh-c29, Invitrogen) or an equivalent volume of solvent control (DMSO) in 1 mL of medium (DMEM/1% PS/10% FBS) for 12 h.

### Flow cytometry analysis of peritoneal macrophages

Mice were disinfected with 70% ethanol and injected with 5 mL of ice-cold PBS (with 3% FBS) into the peritoneal cavity using a 27 G needle. The peritoneum was gently massaged to dislodge any attached cells into the PBS solution and the fluid was collected. The isolated cells were centrifuged at 4°C at 1,500 rpm for 5 min. The staining procedure was described in ref 21. In brief, the cell pellets were incubated with Fixable Viability Dye (Cat. 65-0866-14, ThermoFisher Scientific, 100 μL/mL) for dead cells, and anti-CD16/32 mAbs (Cat. E-AB-F0997A, Elabscience, 50 μL/mL) to block Fc receptors, followed by staining with fluorescent antibodies against cell surface molecules for 1 h at 4°C, including antibodies of anti-F4/80 (Cat. E-AB-F0995C, Elabscience, 50 μL/mL), anti-CD11b (Cat. E-AB-F1081J, Elabscience, 50 μL/mL), and anti-CD86 (Cat. E-AB-F0994E, Elabscience, 50 μL/mL). For intracellular marker staining, cells were fixed and permeabilized using a Fixation/Permeabilization kit (Cat. 00-5223-56, Invitrogen, 100 μL/mL), and stained for anti-CD206 antibody (Cat. E-AB-F1135D, Elabscience, 100 μL/mL) at 4°C for 1 h. The prepared cells were analyzed by a flow cytometry machine (Beckman Coulter CytoFLEX-LX) to determine the cell populations of macrophages. Debris, dead cells, and adherent cells were excluded from the analysis (Figure S11A).

### Adoptive cell transfer experiment

Peritoneal cells were collected from infected WT or *Cnlp*^−/−^ donor mice at week 12 post-infection. The obtained cells were cultured in DMEM with 10% FBS and 1% PS for 4 h, and then the adherent cells on the 12-well plates (TC non-treated) were collected using 2 mL gentle cell dissociation reagent for 5 min. The cell live ratio was accessed using the Trypan Blue staining method. The harvested peritoneal macrophages (1  ×  10^6^ cells/mouse, >90% live rate) were intraperitoneally transferred into recipient mice every 4 days for 3 times using a 1 mL syringe fitted with a 29 G needle. Feces were collected every 4 days for accessing bacteria abundances using qPCR. The peritoneal macrophages isolated from naive and infected mice were also subject to RNA-Seq sequencing and CRAMP measurement.

To demonstrate that peritoneal macrophages could migrate to the colonic lamina propria, we employed a FITC-labeling method as previously reported.^22^ Specifically, a FITC-dextran (Cat. ST2947, Beyotime) uptake assay was performed by seeding purified peritoneal macrophages (0.5  ×  10^5^ cells/well) in 12-well plates. FITC-dextran was then added at a final concentration of 0.5 mg/ml, and the plates were incubated for 45 min at both 4°C and 37°C. After incubation, any unbound FITC-dextran was thoroughly washed away, and the macrophages were detached with 2 ml of gentle cell dissociation reagent. Next, 2  ×  10^6^ labeled macrophages were transferred intraperitoneally into recipient mice. Twelve hours later, lamina propria mononuclear cells from the colonic lamina propria were isolated, followed by flow cytometry analysis to determine the median fluorescence intensity (MFI).

### Immunofluorescence staining

Total peritoneal cells were fixed in freshly made 4% paraformaldehyde at room temperature for 6 h and embedded in paraffin. Sections were washed twice in xylene followed by rehydration in decreasing concentrations of ethanol. Antigen retrieval was conducted to unmask epitopes by boiling in 10 mm citrate for 15 min followed by washing in PBS. Non-specific binding was blocked by 3% bovine serum albumin (BSA). The prepared sections were then stained overnight at 4°C using different primary antibody cocktail settings for CRAMP (Cat. sc-166,055, Santa Cruz Biotechnology, 1:100 dilution), F4/80 (Cat. GB3373, Servicebio, 1:500 dilution), CD206 (Cat. GB3497, Servicebio, 1:200 dilution), and CD86 (Cat. GB5630, Servicebio, 1:100 dilution), respectively. Appropriate goat anti-rabbit (Cat. GB1303, Servicebio, 1:300 dilution) or goat anti-mouse (Cat. GB1301, Servicebio, 1:300 dilution) secondary antibodies labeled with fluorescence were then applied to slides for 50 min at room temperature in the dark in a humidified chamber. Slides were washed and mounted with DAPI (Cat. G1012, Servicebio, 2 μg/mL). The final step involves mounting the sections using an anti-fading agent to preserve fluorescence signal integrity during microscopic examination. Immunofluorescence signaling was captured using an inverted fluorescence microscope (Nikon, Nikon Eclipse C1) and a scanner machine (3DHISTECH, Pannoramic MIDI).

### Fecal microbiota transplantation (FMT)

For the fecal microbiota transplantation (FMT) experiments, recipient mice were gavaged with a 200 μL cocktail of antibiotics (ABX) for 5 days. The ABX cocktail included ampicillin (Cat. A9518-25 G-9, Sigma-Aldrich, 5 g/L), vancomycin (Cat. V2002-1 G, Sigma-Aldrich, 2.5 g/L), gentamicin (Cat. G1914-5 G, Sigma-Aldrich, 5 g/L), neomycin (Cat. N6386-25 G, Sigma-Aldrich, 5 g/L), and metronidazole (Cat. M3761-25 G, Sigma-Aldrich, 5 g/L). The efficiency of this ABX cocktail has been previously proved^23^ and was assessed in this study by evaluating the total bacterial load in feces through 16S rRNA denaturing gradient gel electrophoresis and qPCR targeting the 16S rRNA gene.^10,24^ The FMT procedure was started 2 days after the ABX treatment procedure. Fresh fecal pellets were collected from donor mice, diluted in cold sterile PBS (100 mg/mL), filtered using a double layer of 80-mesh brass screen filter, and orally administered to the recipient mice daily for 1 week. The similarities of the gut microbiomes between donor and recipient mice were evaluated using 16S rRNA sequencing analysis (Figure S9B and S9C).

For FMT experiments that used naive and infected mice as donors, gut microbiota was transferred into healthy recipient mice from the mice at week 12 post-infection. After 4 weeks post FMT procedure, the recipient mice were processed to flow cytometry analysis of colonic Treg or being infected with *Emu* and evaluated for the parasite burden at week 12 post-infection. For the FMT experiments using CRAMP-treated mice as donors, the donor mice were pre-treated with CRAMP (i.v., 4 mg/kg body weight) and vehicle control (PBS) every 4 days for 3 times. The FMT was performed daily from day 4 to 10 post-CRAMP treatment.

### Lymphocyte isolation and flow cytometry analysis

The lymphocytes of intestinal lamina propria were isolated from the colon tissue of the mice using the method previously reported.^10^ Briefly, colon tissues were dissected from mice, opened longitudinally, and the gut contents, adipose tissues, and colonic patches were meticulously removed. Subsequently, the tissues were cut into small sections and thoroughly washed with ice-cold PBS. Tissues were incubated at 37°C in PBS supplemented with 1 mm DTT and 30 mm EDTA. Following this step, colonic epithelial cells were collected for qPCR analysis. Then, the residual tissue fragments were digested for 80 min at 37°C by collagenase type IV (Cat. 14019, Gibco, 200 U/mL) and DNase I (Cat. D8071–25, Solarbio, 150 μg/mL) in 5 mL RPMI1640 medium (Cat. C5500BT, Gibco). The cell suspension was then centrifuged in a Percoll gradient at 4°C at 2,000 rpm for 20 min, and lymphocytes were collected from the 40% to 80% interphase. The isolated 2 × 10^6^ colonic lymphocytes were used for the flow cytometry analysis, as described in ref 10. Briefly, the cells were stained with antibodies of Fixable Viability Dye (Cat. 65-0866-14, eBioscience, 1 µL/mL) for dead cells, and anti-CD16/32 mAbs (Cat. E-AB-F0997A, eBioscience, 10 μg/mL) to block Fc receptors, followed by staining with antibodies against surface markers, including anti-CD3 (Cat. E-AB-F1013J, eBioscience, 50 μL/mL), and anti-CD4 (Cat. E-AB-F1097S, Elabscience, 50 μL/mL). For intracellular markers, cells were fixed and permeabilized using a Fixation/Permeabilization kit (Cat.00-5123-43, Invitrogen, 1:3 dilution) and stained for anti-FoxP3 (Cat, E-AB-F1238D, Elabscience, 50 μL/mL) and anti-Helios (Cat. 11-9883-82, eBioscience, 2.5 μg/mL) at 4°C for 1 h. The results were analyzed on a flow cytometer (Coulter CytoFLEX-LX, Beckman) (Figure S11B).

### QPCR measurement for gene expression

The cDNA for mRNA of total RNA for the tissues or cells was synthesized with OligoDT primers using GoScript Reverse Transcription System (Cat. A5001, Promega) kit with Tm = 25°C. The expression levels of the genes *Cramp*, *Tlrs*, *Vdr*, *Cyp27b1, IL-10*, and *Foxp3* in each sample were measured using qPCR with primers described in Table S1. The qPCR procedure was carried out using the SYBR Green-based method of GoTaq® qPCR Master Mix Kit (Cat. A6002, Promega) according to the manufacturer’s recommendation (Tm = 60°C) on a Real-Time PCR system (Applied Biosystems 7500, Thermo Fisher Sci). The relative expressions were calculated using the 2^−ΔΔ^ CT method and the housekeeping genes, including *Rps13* for BMDM and *B2M* for other cells or tissues (Table S1), were used for internal control to normalize the expression of the target genes.

### Vitamin D supplement in diet

Two weeks before infection, the mice were started to be fed dietary food (Cat. 22922, Keao Xieli Feed Co., Ltd.) supplemented with either a high dose of vitamin D (10,000 IU/kg) or a normal dose (1,000 IU/kg). The mice were intraperitoneally injected with 2,000 *Emu* protoscoleces suspended in 200 μL of PBS or vehicle control. Following a 12-week post-infection period, the mice were euthanized, and serum and fecal samples were collected.

### *Trichinella spiralis* and human tapeworm data

The *Trichinella spiralis* ‘Henan’ strain (ISS534) was utilized in this study. Muscle larvae (ML) were isolated from infected C57BL6J mice using the conventional artificial digestion method, as previously described.^25^ Mice were intragastrically administered 500 ML (diluted in 0.2 mL PBS) or the same volume of PBS as vehicle control. Fecal samples were collected starting from week 6 to 8 post-infection. Following euthanasia, fecal, serum, and colonic tissue samples were harvested for subsequent analyses. Fecal samples for the patients infected with *Taenia asiatica* (*n* = 9) and healthy individuals (*n* = 11) were randomly selected from the Dali cohort previously studied^26^ in our lab to assess the correlation between the levels of *Lactobacillus* and LL-37 in human feces.

### 16S rRNA sequencing and data analysis

The extracted DNA from the colon contents of mice was qualified for its concentrations and integrity using Qubit 2.0 Fluorometer and Agilent 2100 bioanalyzer. The PCR primers targeting either the 16S rRNA V4 region or V3-V4 region (Table S1) were used for amplification and the PCR products were used for constructing libraries by TruSeq® DNA PCR-Free Sample Preparation Kit (Cat. FC-121-3001/3003, Illumina) as per the manufacturer’s protocol. The libraries are sequenced on a NovaSeq 6000 system (Illumina).

The raw reads from each sample were merged into raw tags using FLASH (v1.2.11)^27^ (–min-overlap = 10). The raw tags were filtered for reverse primers by Cutadapt (v3.3)^28^ (–minimum-length = 20) and low-quality reads (the bases with Qphred ≤19 accounting for >50% in the read) by fastp^29^ (–qualified_quality_phred = 19, –unqualified_percent_limit = 50) (v0.23.1). Chimeric sequences were removed by aligning the tags against the Silva 138.1 database using Vsearch (v2.16.0).^30^ The valid data was then processed using QIIME2 (v-QIIME2–22202)^31^ with DADA2 to denoise the data. Species annotation was performed using QIIME2, with the Silva 138.1 database. Alpha Diversity and Beta Diversity were calculated using Phyloseq (v1.3.2.0) and community differential abundance analysis was performed using LEfSe (v1.0.8)^32^ with default parameters.

### RNA-seq sequencing and data analysis

The murine peritoneal macrophages were collected from naive mice and mice infected with *Emu* for 6 weeks. The quality of the extracted total RNA was checked by Qubit 2.0 Fluorometer and Agilent 2100 bioanalyzer. Sequencing libraries of mRNA were constructed using Fast RNA-seq Lib Prep Kit V2 (Cat. RK0306, ABclonal Technology) and sequenced by Illumina X Plus platform. Raw data were trimmed for adaptors and filtered for low-quality reads (the bases with Qphred ≤5 accounting for >50% in the read) by fastp (v0.23.1), resulting in at least 5.9 Gb clean reads for each sample. Hisat2 (v2.0.5)^33^ was used to align the reads to the reference genome (mus_musculus_c57bl6nj_c57bl_6nj_v1_toplevel) with default settings. The FPKM values were calculated using FeatureCounts (1.5.0-p3)^34^ and the differentially expressed genes (q-value < 0.05) were identified using DESeq2 (v1.20.0).^35^ Gene set enrichment analysis (GSEA) (v4.3.3)^36^ was performed using GSEA software (-permute phenotype -metric Signal2Noise) with a gene list ranked by log_2_-transformed fold change between naive and infected samples.

### Quantitative analysis of short-chain fatty acids (SCFAs)

Fecal samples from mice were collected and prepared for measurements of short-chain fatty acids, including acetic acid, propionic acid, butyric acid, pentanoic acid, hexanoic acid, isobutyric acid, and isovaleric acid by the GC-MS system (Agilent 7890B-5977B), as previously described.^10^ Data was processed using Agilent’s MassHunter Quantitative Analysis software (vB.07.01), which automatically identified and integrated ion fragments with manual verification to confirm accuracy. A linear regression standard curve was constructed by plotting the peak areas against the known concentrations, facilitating the quantitative analysis of the short-chain fatty acids present in the fecal samples based on their spectral peak areas.

### Statistical analysis

Statistical analyses were performed using R software (v4.2.0) and GraphPad Prism (v9.0.2). Data differences between the two groups were assessed using the two-sided Student’s t-test and Wilcoxon's rank-sum test with a 95% confidence interval. The two-way repeated analysis of variance (ANOVA) followed by Sidak’s posttest was used for multiple-group comparisons for experimental data. The *p*-values in multiple hypothesis testing for the microbiome or RNA-seq data were corrected using the Benjamini–Hochberg procedure (*FDR*). PERMANOVA analysis was used to determine the *p* value in PCoA. Spearman’s correlation coefficient was used for correlation analysis. A *P* or *FDR* value less than 0.05 was considered statistical significance.

## Results

### *E.* *multilocularis* infection induces *Lactobacillus* expansion at an early infection stage

**Figure 1.**
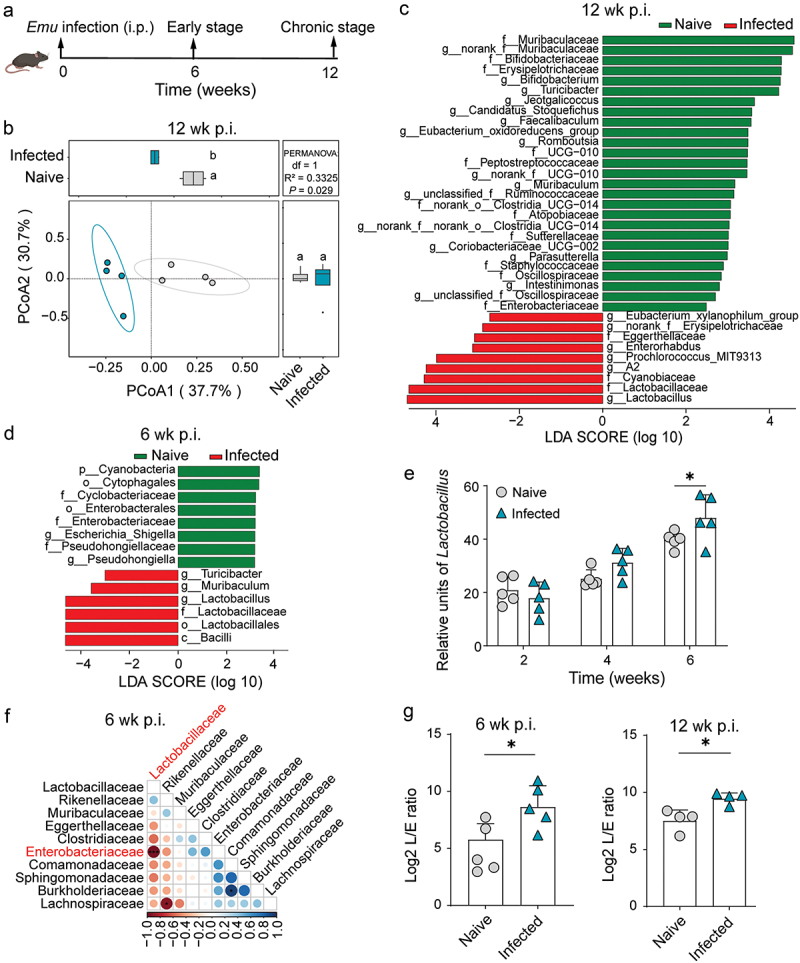
Intestinal lactobacilli bacteria are increased at the early stage of *Emu* infection. (a) Schematic representation of treatment and sampling for the mice intraperitoneally infected with *Emu* infection (i.p., *n* = 2,000 protoscoleces) and naive mice. (b) The PCoA with 95% confidence ellipse for the gut microbiome (16S rRNA sequencing) based on Bray–Curtis distance from the mice at 12 weeks post-infection. (c and d) Linear discriminant analysis Effect Size (LEfSe) analysis between the gut microbiome of colon content for naive and infected mice at 12 weeks (c) and 6 weeks post-infection (d). The effect size of each differentially abundant feature is estimated by linear discriminant analysis (LDA) in LEfSe. Only the taxa with LDA score (log 10) >2 are shown. (e) qPCR analysis of *Lactobacillus* in feces across time post-infection. The relative units were calculated using a standard prepared with *in vitro* cultured bacteria. (f) Spearman correlation analysis between Lactobacilli and other microbes at the family level. Only the top 10 taxa ranked by *p*-values in the correlation analysis are shown. (g) The log2-ratio of *Lactobacillus* to Enterobacteriaceae (L/E ratio) within the gut of the mice at weeks 6 (left) and 12 (right) post-infection. Data presented as mean ± SD for panels e and g. Results are representative of two independent experiments with 4 (for panels b, c, and left g) or 5 (for panels d, e, f, and right g) mice per group. The *p* values were determined by the PERMANOVA test for panel b, by the two-sided *student’s t*-test for panels e and g, and by the Spearman correlation coefficient analysis for panel f (*p < 0.05, **p < 0.01, ***p < 0.001).

To examine the effect of larval *E. multilocularis* (*Emu*) infection on the host gut microbiome, we sequenced 16S rRNA of colon lumina content from C57BL6 mice at 12 weeks (chronic stage) post-infection (p.i.) (Figure 1(a)). Principal coordinate analysis (PCoA) based on Bray–Curtis distance matrix of amplicon sequence variants (ASVs) revealed that the gut microbial compositions were significantly skewed in infected mice (Figures 1(b) and S1A). As measured by the Shannon index, alpha diversity was significantly reduced (Figure S1B). These results indicate that *Emu* infection markedly affects the gut microbial community. Consistent with previous findings for various helminth infections,^10^ these alterations were indicated by a significant increase of lactobacilli (*Lactobacillus*) (Figure 1(c)).

To investigate whether these changes occurred at an early infection stage, we compared the results to the gut microbiome at 6 weeks p.i. We found that the relative abundance of lactobacilli was already significantly elevated at this time (Figure 1(d)), although the overall composition changes in terms of alpha or beta diversity were not as noticeable as those at 12 weeks p.i. (Figure S1C and S1D). Furthermore, qPCR analysis revealed a gradually increased abundance of *Lactobacillus* after infection (Figure 1(e)). These observations suggest that the mechanisms underlying the changes in the gut microbiota likely start at an early stage after infection.

Notably, accompanied by the early accumulation of gram-positive *Lactobacillus*, the depleted bacteria (Gammaproteobacteria or Cytophagia), including Enterobacteriaceae, Pseudohongiellaceae, and Cyclobacteriaceae, were all gram-negative (Figure 1(d)). Among them, enterobacteria (Enterobacteriaceae) showed the most negative correlation with lactobacilli (Figure 1(f)), resulting in a considerably higher lactobacilli to enterobacteria (L/E ratio) in *Emu*-infected compared to naive mice (Figure 1(g)). Throughout this study, we use the L/E ratio to indicate infection-associated microbiota alterations, as it has been frequently used as a marker for the gut health status of animals.^19^

### CRAMP reshapes the host gut microbiome and promotes *Lactobacillus* growth in helminth infections

**Figure 2.**
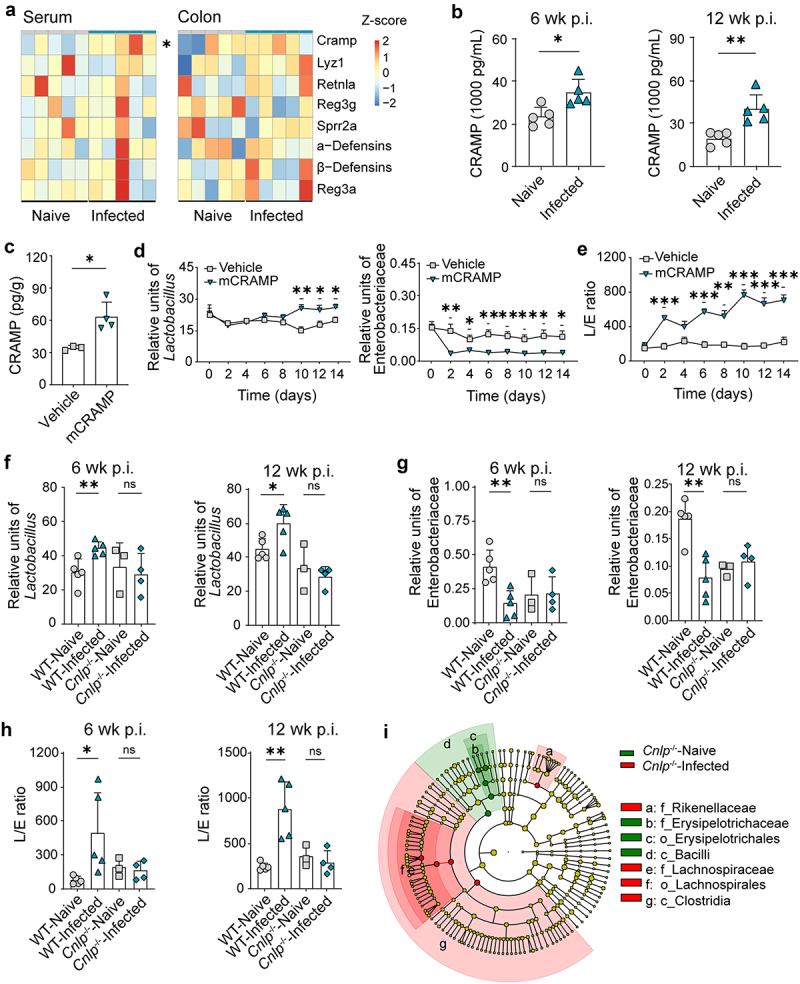
CRAMP drives the expansion of lactobacilli bacteria during *Emu* infection. (a) ELISA measurements of antibacterial peptides (AMPs) in serum and colon tissue in the naive mice and mice infected with *Emu* 6 weeks post-infection. The expression levels were scaled between naive and infection and are shown in Z-score. (b) The protein level of CRAMP in the serum of the mice at the early or chronic infection stage. (c) The increased level of CRAMP in the feces of the mice 2 hours after injection with mCRAMP. (d and e) the abundances of *Lactobacillus* and Enterobacteriaceae (d) and L/E ratio (e) in the feces of the mice injected with mouse CRAMP or vehicle control. qPCR analyses were performed every two days. (f, g, and h) the abundances of *Lactobacillus* (f) and Enterobacteriaceae (g) and the L/E ratio (h) in the feces of the CRAMP-KO mice (*Cnlp*^−/−^) or WT mice after *Emu* infection. (i) The altered taxa in *Cnlp*^−/−^ mice at 12 weeks post-infection. Data presented as mean ± SD for panels b, c, and f-h, and as mean ± SE for panels d and e. Results are representative of at least two independent experiments. *n* = 5 for panels a, d, and e; mouse numbers for other data are shown in each panel. *p* values were determined by the two-sided student’s t-test for panels a-c and f-h, and by two-way repeated analysis of variance (ANOVA) followed by Sidak’s posttest for panels d and e (*p < 0.05, **p < 0.01, ***p < 0.001).

We next sought to explore exactly what promotes the expansion of the *Lactobacillus* population. Since it has been widely reported, irrespective of helminth or host species,^5,6^ we suspected a host response-associated mechanism. The altered ratio of gram-positive to gram-negative bacteria (see above) suggested that antimicrobial peptides (AMPs), which can disrupt the integrity of bacterial membranes,^37,38^ were potentially involved. Using ELISA, we thus screened colonic tissue and blood serum at 6 weeks p.i. for major peptides known for antibiotical activities (Figure 2(a)). Of all detected AMPs, only the level of CRAMP, which targets gram-negative bacteria, in serum, was significantly increased (Figure 2(b)). We further confirmed the only increase of CRAMP at the chronic infection stage (Figure 2(b) and Figure S2A). Although CRAMP expression in the murine colon has been reported to be largely restricted to epithelial cells,^39,40^ we did not find any differences in its expression between intestinal epithelial cells of infected and naive mice (Figure S2B). These results suggest that helminth infection induces increased expression of CRAMP in the circulatory system of mice.

To investigate whether the higher CRAMP level in serum affects *Lactobacillus* fitness, we first determined its presence in the intestinal tract via extra-intestinal injections. We peritoneally or intravenously (data not shown) injected the mice with CRAMP and noted that its fecal concentration was rapidly augmented (Figure 2(c)), indicating that the circulating system-derived AMP had direct contact with the gut microbes. We then went on to test whether CRAMP could regulate the gut microbiota in the context of *Lactobacillus* expansion and L/E ratio. We injected mice with CRAMP every 3 days and observed a rapidly decreasing level of Enterobacteriaceae from day 2 and a gradually increasing level of *Lactobacillus* from day 10, compared to the control group (Figure 2(d)). Therefore, this treatment resulted in a significantly increased L/E ratio (Figure 2(e)). These results demonstrate that circulating CRAMP is indeed capable of regulating *Lactobacillus* and Enterobacteriaceae fitness within the gut.

To further examine the impact of CRAMP signaling on *Lactobacillus* and Enterobacteriaceae growth, we analyzed the levels of these bacteria in *cramp-*knockout mice (*Cnlp*^*-/-*^; deficient in CRAMP expression) and wild-type (WT) littermates. In contrast to WT mice, *Lactobacillus* levels were not raised in the infected *Cnlp*^−/−^ mice compared to naive *Cnlp*^−/−^ mice at either the early or chronic infection stage (Figure 2(f)), and neither was Enterobacteriaceae abundance depleted in the infected vs. the naive *Cnlp*^−/−^ mice (Figure 2(g)). Accordingly, the L/E ratio did not differ between naive and infected *Cnlp*^−/−^ mice (Figure 2(h)). Moreover, PCoA of 16S rRNA sequences revealed only a non-significant shift (*p* >0.05, PERMANOVA test) of the whole microbiome structure in infected *Cnlp*^−/−^ mice even at 12 weeks p.i., with a minimal number of the differentially abundant taxa (*n* = 3 at the genus level) (Figure 2(i) and S3). In summary, these results suggest that CRAMP promotes the expansion of *Lactobacillus* during helminth infection.

We also validated the above findings in other helminth infections, including experimental *T. spiralis* infection in mice and *Taenia* tapeworm infection in humans. Interestingly, we found that *T. spiralis* infection promoted *Lactobacillus* growth in the gut of mice with increased CRAMP levels in serum and gut content at 8 weeks p.i. (tissue-dwelling stage) (Figure S4A-C). Importantly, this trend was not observed in *Cnlp*^*-/-*^ mice (Figure S4D). For intestinal *Taenia* infection, levels of the CRAMP homolog cathelicidin LL-37 were also increased and significantly correlated with increased *Lactobacillus* levels in a cohort of humans with Taeniasis (Figure S4E and S4F). These data corroborate the idea that CRAMP-dependent promotion of *Lactobacillus* is a common mechanism during helminth infections, in line with the observation that expanded populations of this bacterium are frequently reported in various parasite-host systems.^5^

### CRAMP increases *Lactobacillus* fitness by indirectly supporting its ecological competition via inhibiting gram-negative microbes

**Figure 3.**
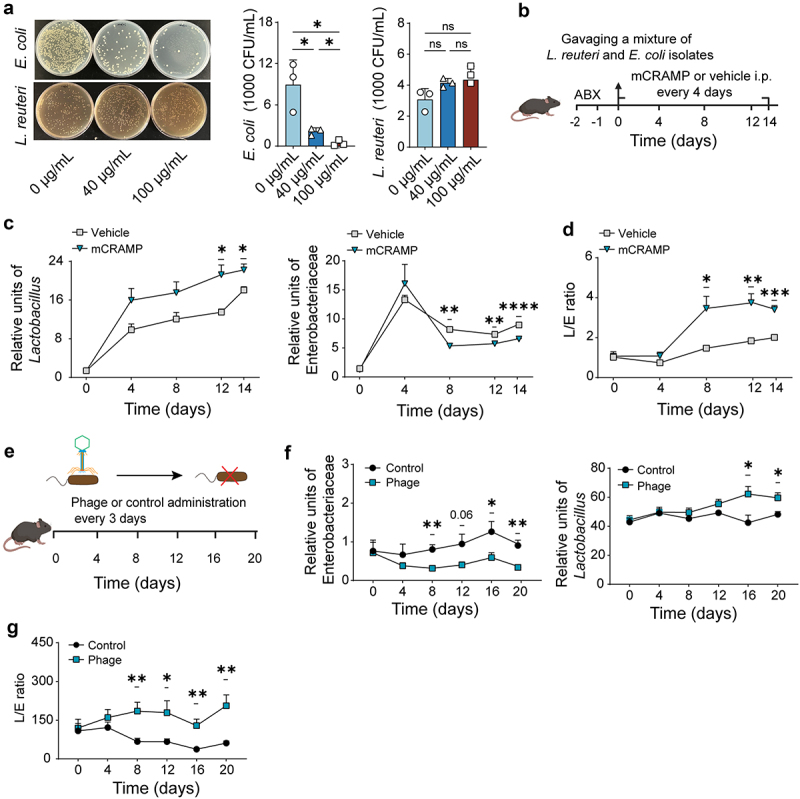
CRAMP supports lactobacilli growth by inhibiting other microbes. (a) The inhibition of CRAMP on the two bacteria isolates. Each of the isolates was incubated with different concentrations of CRAMP for 2 h and the culture was then plated on agar plates for counting. (b) Experiment schema for the competition model in the mice. The mice were treated with CRAMP or vehicle after treatment with an antibiotics cocktail. (c and d) the abundances of *Lactobacillus* (c, left) and Enterobacteriaceae (c, right) and the L/E ratio (d) in the feces of the mice with or without CRAMP treatment. (e) Experimental schema for depleting the *E. coli* isolates in the gut using phage. The mice were gavaged either with phage or control (dead phage) every 3 days. (f and g) the abundances of Enterobacteriaceae (f, left) and *Lactobacillus* (F, right) and L/E ratio (g) in the feces of the mice with or without phage treatment. Data presented as mean ± SE for panels c, d, f, and g. Results are representative of at least two independent experiments. *N* = 3 for panel a; *n* = 4 or 5 for panels c and d; *n* = 7 for panels f and g. *p* values were determined by the two-sided student’s t-test for panel a, and by two-way repeated analysis of variance (ANOVA) followed by Sidak’s posttest for panels c, d, f, and g (*p < 0.05, **p < 0.01, ***p < 0.001,****p < 0.0001).

We sought to gain insights into the mechanisms by which CRAMP influences helminth-induced microbial alterations. The above results show a rapid reduction of Enterobacteriaceae followed by a gradual increase of *Lactobacillus* after CRAMP injection (Figure 2(d)) suggesting that CRAMP indirectly supports the growth of *Lactobacillus* by inhibiting Gram-negative bacteria. To test this hypothesis on a species level, we first determined which *Lactobacillus* and Enterobacteriaceae species expanded during *Emu* infection in the mice, using isolation, identification, and colony-counting verification for strains (Figure S5A). We found that isolates of *L. reuteri* and *E. coli* were abundant and significantly over-represented in the guts of *Emu*-infected mice at either early or chronic stages (Figure S5B). We then examined the inhibition effect of CRAMP through an *in vitro* culturing experiment (Figure 3(a)) and observed that CRAMP efficiently inhibited the growth of *E. coli* in a dose-dependent manner but had almost no effect on the growth of *L. reuteri* (Figure 3(a)).

To test this further, we designed an *in vivo* competition assay by gavaging a mixture of the isolated *L. reuteri* and *E. coli* strains with equal CFUs into mice treated with an antibiotic cocktail (Figure 3(b)). We then injected the mice with either CRAMP or a vehicle control. In mice treated with CRAMP, we observed higher levels of *L. reuteri* (Figure 3(c), left) and reduced levels of *E. coli* (Figure 3(c), right), as well as a higher ratio of *L. reuteri* to *E. coli* (Figure 3(d)) compared to the control mice, suggesting that *Lactobacillus* outcompeted Enterobacteriaceae in the presence of CRAMP.

To further investigate whether the direct inhibition of Enterobacteriaceae would promote *Lactobacillus* within the gut, we selectively depleted *E. coli* using a bacteriophage. We isolated a lytic bacteriophage (hereafter, phage) that specifically targets the *E. coli* isolate (Figure 3(e)) from a brown rat and gavaged this phage or control (dead phage) into mice. The administration of live phage resembled the CRAMP treatment model, in which the level of *E. coli* (Figure 3(f), left) was rapidly reduced and the level of *L. reuteri* (Figure 3(f), right) was promoted, significantly changing the ratio between them (Figure 3(g)). Taken together, our results support the idea that CRAMP likely impacts *Lactobacillus* fitness by directly inhibiting CRAMP-sensitive microbes.

### CRAMP-expressing macrophages expanded in helminth infection promote *Lactobacillus*

**Figure 4.**
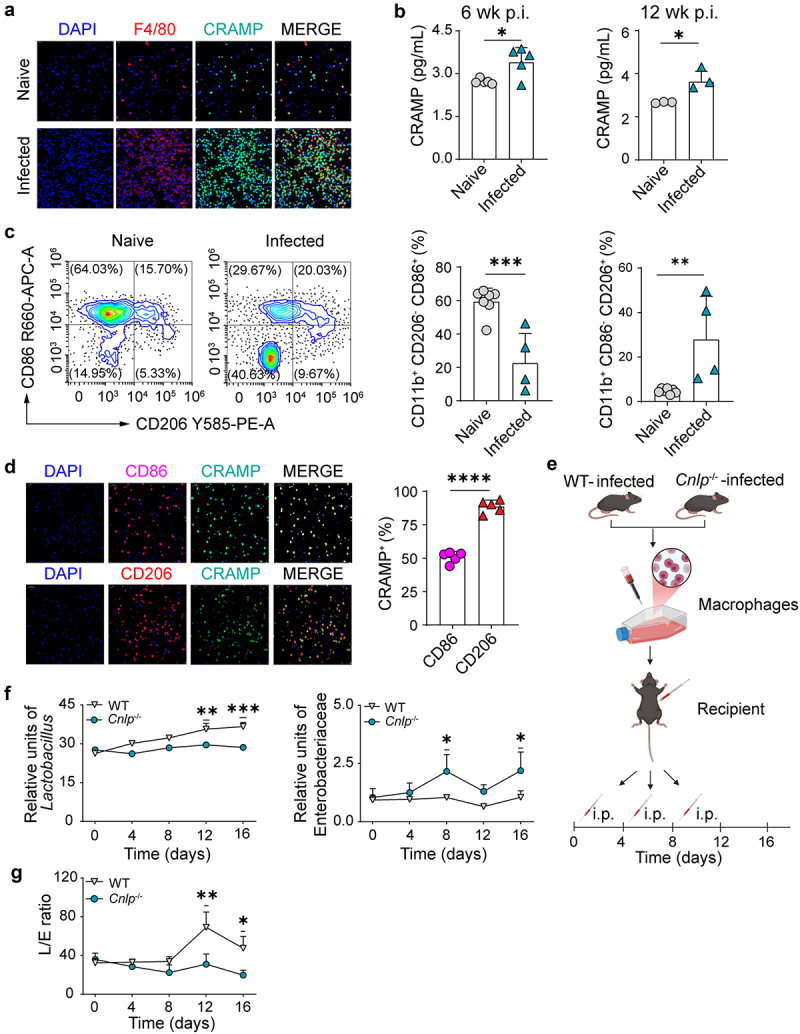
CRAMP-producing macrophages drive lactobacilli expansion. (a) Representative immunofluorescence images of F4/80^+^ cells and CRAMP^+^ cells in the peritoneal macrophages isolated from infected mice (6 wk p.i.). (b) The expression of CRAMP in peritoneal macrophages at different infection stages. (c) Flow cytometry analysis of peritoneal macrophages in naive or infected mice (6 wk p.i.). CD11b^+^CD206^+^ or CD11b^+^CD86^+^ cells were gated from F4/80 + cells. (d) Representative immunofluorescence images of CD86^+^/CD206^+^ cells and CRAMP^+^ cells in the peritoneal macrophages isolated from infected mice (6 wk p.i.). The proportion of CRAMP^+^ cells in CD86^+^ or CD206^+^ cells was counted. (e) Schematic representation of treatments in macrophages adoptive transfer experiments for wild-type (WT) and CRAMP-KO (*Cnlp*^−/−^) mice. Peritoneal macrophages isolated from infected WT or KO mice were purified and transferred into the receptor mice every 4 days. (f and g) the abundances of *Lactobacillus* (f, left) and Enterobacteriaceae (f, right) and the L/E ratio (g) in the feces of the CRAMP-KO mice (*Cnlp*^−/−^) or WT mice after cell transfer were measured using qPCR. Data presented as mean ± SD for panels b-d, and as ± SE for panels f and g. Flow cytometry data are representative of *n* = 4 or 7 mice per group; mouse numbers for other data are shown in each panel. Results are representative of at least two independent experiments. *p* values were determined by the two-sided student’s t-test for panels b-d, and by two-way repeated analysis of variance (ANOVA) followed by Sidak’s posttest for panels f and g (*p < 0.05, **p < 0.01, ***p < 0.001, ****p < 0.0001).

Next, we investigated what the sources of CRAMP expression during helminth infection could be. The high level of CRAMP in blood serum suggests a major source from peripheral circulation. Beside epithelial cells, murine CRAMP is mainly expressed by peripheral neutrophils and monocytes.^41,42^ Using a peripheral blood cell isolation method, we found that mononuclear cells are a major source of circulating CRAMP, while granulocytes also express CRAMP to some extent (Figure S6A). We further confirmed this observation by immunofluorescence staining of peritoneal immune cells and found that in *Emu*-infected mice at 6 weeks p.i., CRAMP is highly expressed in the F4/80^+^ macrophages (Figure 4(a)). We thus isolated the peripheral macrophages, which highly proliferated during infection, and detected CRAMP using ELISA and qPCR. We found that CRAMP production capability in the macrophages was markedly higher in infected mice (Figure 4(b) and Figure S6B). Our findings suggest that macrophages are a marked source of circulating CRAMP during *Emu* infection.

To investigate this further, we queried the different macrophage phenotypes occurring during *Emu* infection for CRAMP by analyzing the ratio of peritoneal macrophage phenotypes using flow cytometry (Figure 4(c)). As expected, CD206^+^ macrophages prevailed in infected mice (% in F4/80^+^CD11b^+^ cells) (Figure 4(c)), while CD86^+^ macrophages dominated in naive mice (% in F4/80^+^CD11b^+^ cells) (Figure 4(c)). In infected mice, immunofluorescence staining revealed a high proportion of CD206^+^ macrophages with CRAMP expression (Figure 4(d)), suggesting that alternatively activated macrophages (M2; CD206^+^) are a prominent source of CRAMP during helminth infection.

Next, we further validated whether macrophage-derived CRAMP signaling during helminth infection promotes *Lactobacillus* expansion. We isolated post-infection peritoneal macrophages from WT and *Cnlp*^−/−^ mice and adoptively transferred them into acceptor mice (Figure 4(e)). First, using the FITC dextran-labeling method, we determined that the transferred peritoneal macrophages using i.p. injection could be trafficked to colon lamina propria (Figure S7). We found that *Lactobacillus* levels were significantly higher in recipient mice transferred with macrophages from WT mice than in recipient mice transferred with those from *Cnlp*^−/−^ mice (Figure 4(f), left), concomitant with a reduced level of Enterobacteriaceae (Figure 4(f), right). This resulted in a higher L/E ratio in mice accepting WT macrophages compared to that in mice transferred with *Cnlp*^−/−^ macrophages (Figure 4(g)). Collectively, these results suggest that antibacterial programs from macrophages could promote *Lactobacillus* fitness during *Emu* infection.

### Secretory components of helminths drive CRAMP expression in macrophages through dietary 1,25-(OH)_2_D_3_-regulated TLR2 signaling pathway

**Figure 5.**
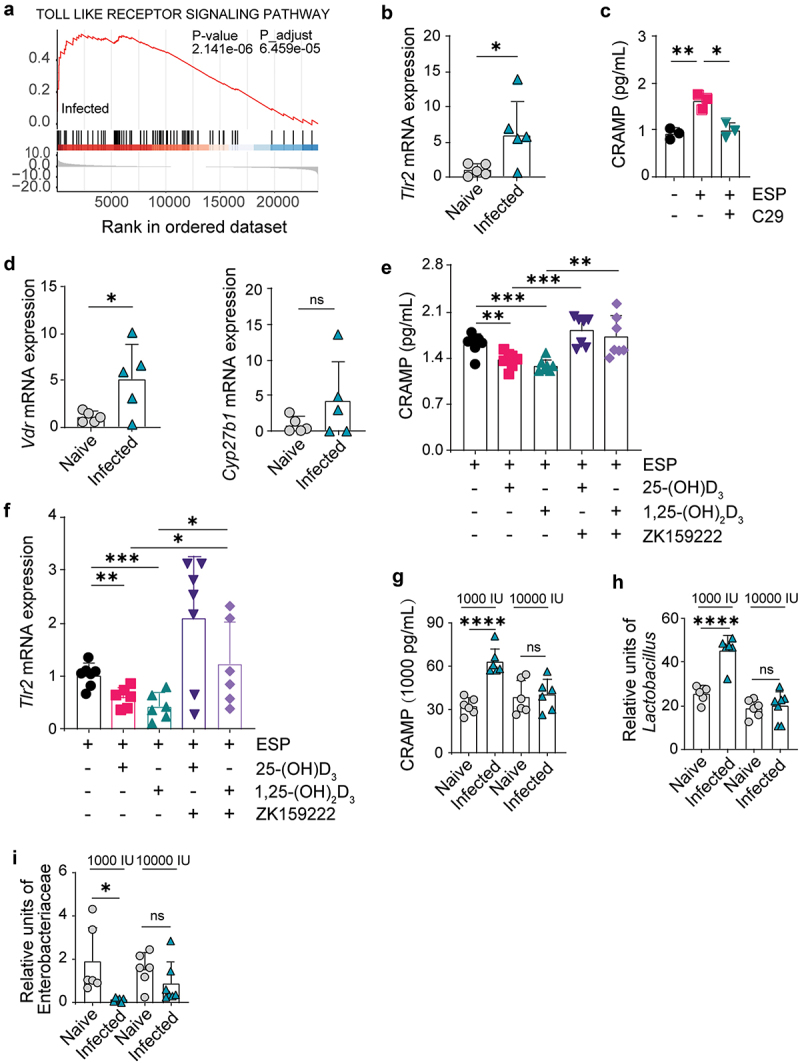
Helminth secretory components drive CRAMP expression through dietary vitamin D3-regulated TLR2 signaling in macrophages. (a) Gene set enrichment analysis of the genes in the peritoneal macrophages. Genes were ranked by the log2-transformed fold-change of expression levels between naive and infected mice. (b) The expression level of *Tlr2* in the peritoneal macrophages isolated from the mice at 6 weeks post-infection. (c) CRAMP expression in bone marrow-derived macrophages (BMDM) after treatment with excretory/secretory products (ESP) of larval *Emu*. The *Tlr2* inhibitor C29 (100 µM) was tested for its role in regulating CRAMP. (d) qPCR analysis of the expression level of *Vdr* and *Cyp27b1* in the peritoneal macrophages isolated from the mice at 6 weeks post-infection. (e and f) the expression of CRAMP (e) or *Tlr2* (f) in BMDM after treatments with ESP with or without the presence of vitamin D (25-(OH)D_3_ or 1,25-(OH)_2_D_3_). The vitamin D receptor inhibitor (ZK9222) was tested for its effect on inhibiting *vdr* in regulating CRAMP or *Tlr2* expression. (g) The protein level of CRAMP in the serum of the mice fed a diet with normal (1,000 IU) or high dose (10,000 IU) vitamin D supplement in ELISA analysis. (h and i) the abundance of *Lactobacillus* (h) and Enterobacteriaceae (i) in the feces of the mice fed a diet with normal or high dose vitamin D supplement. qPCR method was used for the measurement. Data presented as mean ± SD, with mouse numbers indicated in each panel. Results are pooled from two independent experiments for panels e and f. *p* values were determined by the two-sided student’s t-test for panels b-i (*p < 0.05, **p < 0.01, ***p < 0.001, ****p < 0.0001).

To determine the signaling pathway that boosts CRAMP expression in macrophages during *Emu* infection, we analyzed the transcriptomes of the peritoneal macrophage*s* of infected vs. naive mice at 6 weeks p.i., using RNA-seq. Gene set enrichment analysis (GSEA) showed that the Toll-like receptor (TLR) signaling pathway is among the most enriched in the macrophages of infected mice (Figure 5(a)). Although the regulation of CRAMP in macrophages via activation of TLR1/2 has been well studied for bacterial infections (e.g., *Mycobacterium tuberculosis*) in humans,^43,44^ it remains poorly investigated in mice, particularly for helminth infections. We could confirm this observation using qPCR and found that *Tlr1*, *Tlr2*, and *Tlr6* (forming TLR1/2 or TLR2/6 heterodimers) had increased activity in infected mice (Figure 5(b) and Figure S8). We then investigated the role of TLR2-related pathways in regulating CRAMP expression in murine bone marrow-derived macrophages (BMDM) *in vitro* and noted that excretory-secretory product (ESP) collected from the *Emu* cysts could significantly promote CRAMP expression in BMDM (Figure 5(c)). This induction was blocked when TLR2 inhibitor (C29) was added to the BMDM culture (Figure 5(c)). These results suggest that ESP-provoked CRAMP expression is regulated via TLR2 activation.

It has been shown that TLRs upregulate LL-37 expression via a vitamin D3-dependent pathway in primates.^43,44^ However, mice lack the key downstream component, vitamin D response element (VDRE), in their *camp* promoters.^44^ Unexpectedly, our results revealed an upregulation of *Vdr* accompanying *Tlr2* overexpression during *Emu* infection in mice (Figure 5(d)). We also observed very low but detectable expression of the enzyme *Cyp27b1*, which hydroxylates 25-(OH)D_3_ to the active form 1,25-(OH)_2_D_3_ (Figure 5(d)). This is in line with the previous studies showing that CD8^+^ T cells rather than macrophages are the major source of *Cyp27b1* expression.^45^ This observation prompted us to investigate the role of the vitamin D_3_ pathway in regulating ESP-induced CRAMP expression in the presence of helminth ESP. We measured the levels of CRAMP and *Tlr2* in BMDM with an additional 25-(OH)D_3_ or 1,25-(OH)_2_D_3_ supplement. In contrast to human macrophages, our results showed that vitamin D_3_ suppresses the expression of CRAMP (Figure 5(e)) and *Tlr2* (Figure 5(f)) in mice. This downregulation was blocked when we added a VDR antagonist (ZK9222), suggesting that the effect of vitamin D_3_ on the TLR2-CRAMP pathway is VDR-dependent (Figure 5(e,f)).

The above results suggest that vitamin D_3_, as an important micronutrient for the host, might be vital in regulating helminth-elicited gut microbiota changes in mice. To test this hypothesis further, we fed naive or infected mice daily with different vitamin D_3_-supplemented diets (normal dose and high dose). We observed the increase of CRAMP in the blood serum (Figure 5(g)), the expansion of *Lactobacillus*, and the reduction of Enterobacteriaceae were fully blocked in infected mice fed with a high dose of vitamin D_3_ (Figure 5(h,i)) but remained the same in mice fed with a normal dose of vitamin D_3_ (Figure 5(g–i)). This highlights the importance of dietary vitamin D_3_ in regulating *Lactobacillus* expansion during helminth infection.

### CRAMP signaling determines gut microbiota-mediated suppressive immunity and helminth survival

**Figure 6.**
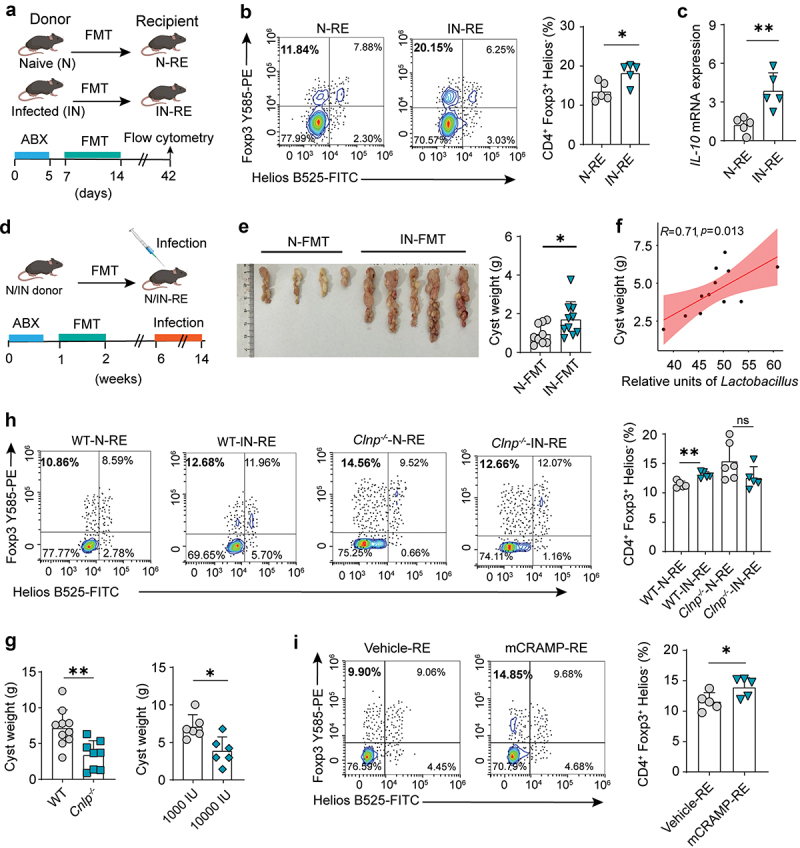
CRAMP signaling regulates gut microbiota-mediated suppressive immunity in helminth infection. (a) Schematic representation of fecal microbiota transplantation (FMT) regime using naive and infected mice as donors. Recipient mice (RE) were pretreated with antibiotics (ABX) cocktails for 5 days, followed by an FMT regime of 1 week from naive donors (N) or infected donors (IN) around 12 weeks p.i. (b) The proportion of Treg cells in lamina propria of the recipient mice with FMT. The colonic CD4^+^Foxp3^+^ Helios^−^ Treg cells were gated in the CD3^+^CD4^+^ cell population for the experiment shown in panel a. (c) qPCR analysis of the expression level of *IL-10* in the colon tissue. (d) Schematic representation of the experiment with FMT and follow-up infection. The mice were transplanted with the fecal microbiota by a 1-week-FMT regime from naive donors (N) or infected donors (IN) at 12 weeks p.i. Four weeks later, the recipients were infected with *Emu*. (e) The parasite burden in the recipient mice in the experiment shown in panel d. (f) The Spearman correlation between cyst weight and the abundance of *Lactobacillus* for infected mice at 12 weeks p.i. (g) The parasite burden in WT and CRAMP-KO mice (left) and in vitamin D-treated mice (right). (h) The proportion of treg cells (CD4^+^Foxp3^+^Helios^−^) in the CD3^+^CD4^+^ T cell population for the recipient mice in the FMT experiment with WT and *Clnp*^−/−^ mice as donors. The donors were either mice having been infected with *Emu* for 3 months (WT-IN or *Cnlp*^−/−^-IN) or naive mice (WT-N or *Cnlp*^−/−^-N). (i) The proportion of treg cells in the CD3^+^CD4^+^ T cell population for the recipient mice in the CRAMP treatment experiment. FMT was performed using donor mice that were treated with CRAMP (i.v., 4 mg/kg/day for 4 times) or vehicle. Data presented as mean ± SD, with mouse numbers indicated in each panel. Results are representative of two independent experiments. *p* values were determined by Spearman correlation coefficient analysis for panel f and by the two-sided student’s t-test for other panels (*p < 0.05, **p < 0.01, ***p < 0.001).

Altered gut microbiota during helminth infection has been linked to host immunosuppression, marked by Treg expansion.^9,10^ Next, we investigated the role of CRAMP signaling in gut microbiota-mediated immunoregulation during helminth infection. In this model, we first confirmed the role of gut microbes in increasing Treg cell levels through fecal microbiota transplantation (FMT) experiments (Figure 6(a)). Our results showed that gut microbiota transplanted from *Emu*-infected mice induced higher levels of colonic Foxp3^+^ Treg cells (Figure 6(b)) and *IL-10* expression (Figure 6(c)) than gut microbiota transplanted from naive mice, suggesting that *Emu* infection-induced gut microbiota promotes suppressive immunity. In line with this, we found that the fecal short-chain fatty acids (SCFAs), which are well-known Treg inducers, were elevated in the infected donor mice compared to the naive donor mice (Figure S9A). To examine the effect of the transplanted gut microbiota on the parasite burden, we infected recipient mice with *Emu* after FMT. The parasite burden was significantly higher for the mice receiving gut microbiota from infected mice than for the mice receiving gut microbiota from naive donor mice (Figure 6(d,e)). Since lactobacilli can promote the persistence of helminth infection through immunoregulation (e.g., Treg enhancement) in other infection systems,^15,16,46^ we further studied the role of our *Lactobacillus* isolate in immunoregulation. We found that expansion of the *L. reuteri* population led to an increase of *Foxp3* level at the lamina propria (Figure S10), suggesting that the species is capable of regulating Treg differentiation. Of note, we observed that in *Emu*-infected mice, the *Lactobacillus* level in the gut significantly correlated with parasite burden (Figure 6(f)). Taken together, these results show that infection-induced changes of the gut microbiota promote suppressive immunity and parasite survival.

Our data consistently suggest that during *Emu* infection, CRAMP signaling influences bacteria-dependent suppressive immunity by shaping gut microbial composition. In line with this, we found that the parasite burden in *Cnlp*^−/−^ mice or mice treated with high-dose vitamin D, which is deficient of or reduced in circulating CRAMP, was significantly decreased (Figure 6(g)). To test this hypothesis, we performed FMT experiments, using naive or infected WT and *Cnlp*^−/−^ mice as donors. In contrast to that of WT littermate mice, gut microbiota transferred from *Cnlp*^−/−^ donor mice failed to promote Treg (CD4^+^Foxp3^+^Helios^−^) expansion at the colonic lamina propria (Figure 6(h)) of the recipients. Finally, we validated our findings by using the mice that had been directly injected with CRAMP intraperitoneally, as donor mice in FMT. We then investigated the role of their altered gut microbiota in inducing Treg expansion. For the mice that received gut microbiota from donor mice with CRAMP treatment, Treg cell (CD4^+^Foxp3^+^Helios^−^) levels were significantly increased compared to those in the mice receiving gut microbiota from vehicle-treated donor mice (Figure 6(i)). This suggests that after CRAMP treatment, the capability of the gut microbiota to regulate the mucosal immune response is strengthened. Collectively, our data highlight the important role of CRAMP signaling in mucosal immunoregulation during helminth infection.

## Discussion

**Figure 7.**
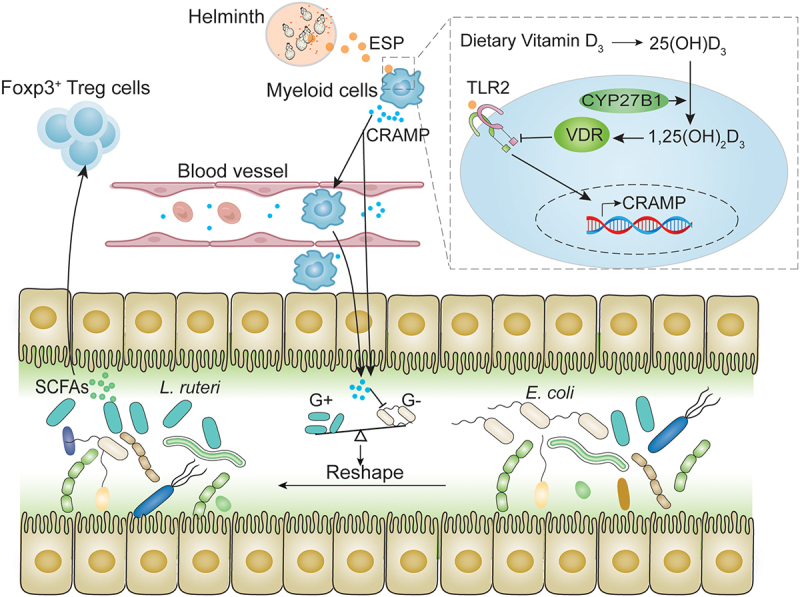
The proposed mechanism in this study. Our results suggest that the CRAMP produced from myeloid cells in helminth infections, particularly macrophages, reshapes the gut microbial community and promotes lactobacilli by inhibiting gram-negative (G-) bacteria and providing ecological niches for gram-positive bacteria (G+). Through this regulation, the gut microbiome and metabolome (e.g., short-chain fatty acids [SCFAs]) are reprogrammed to enhance host Foxp3^+^ Treg cell expansion and parasite survival. The CRAMP production in macrophages is boosted via toll-like receptor signaling by helminth’s excretory/secretory products (ESPs), which could be sufficiently tuned down by dietary vitamin D through its receptor and cyp27b1. The primary source of CRAMP in this model remains to be determined; potential sources include peripheral macrophages (e.g., peritoneal macrophages) that either release CRAMP into the bloodstream, or migrate into the intestine site, and tissue-resident macrophages that are locally activated by circulating helminth ESPs.

With a view to the ‘holobiont’ concept, helminths have evolved a unique interchange with their hosts, including finely tuned molecular adaptations that influence host signaling pathways to ensure the parasites’ persistence.^3^ The most important aspects of this interchange are the manipulation of host immunity and gut microbiota. Recent studies have linked these two aspects by showing that the altered gut microbiome contributes to the immunoregulation triggered by the helminths.^9,10,12,15,47^ This highlights the crucial role of gut microbiota in developing treatments against parasites as well as helminth-based therapies for autoimmunity-related diseases. However, the exact mechanisms that are at work at the interface between helminths and gut microbes remain poorly understood. In this study, we have revealed a mechanism that explains that complex mutualistic relationship by showing how the host’s innate immunity influences the gut microbiota and subsequent suppressive responses (Figure 7).

We have shown that during helminth infection, CRAMP signaling in macrophages causes an expansion of the lactobacilli populations in the host’s gut. Our preliminary investigation of other helminth-host systems, including *T. spiralis*-mouse and *Taenia*-human, further supports the crucial role of CRAMP signaling in controlling lactobacilli populations. However, it remains to be determined whether this pathway is responsible for changes in gut microbiota composition in yet other helminth infection systems and whether it also affects other microbes. In particular, the expansion of *Lactobacillus* bacteria does not consistently correlate with a decrease in Enterobacteriaceae or *E. coli*. For example, in infections caused by *T. muris*, *Lactobacillus* can bloom regardless of whether *E. coli* abundance changes.^48,49^ Since the relationship between helminth, the host, and its gut microbiota is highly complex, there are likely some variations in mechanisms underlying changes to the microbiome. Physical interactions, infection sites, changes in gut pH, competition for nutrients/ecological niches in the host gut, and secreted molecules from parasites or the host are likely to act in the interplay between helminths and microbiota and could at least partially affect the regarding compositions.^50^ Also, other AMPs derived from intestinal epithelium, such as Sprr2a and Retnlb, which are boosted by type 2-promoting cytokines, can affect the host microbiome, as has been reported for *H. polygyrus* ^51^ and *N. brasiliensis* ^47^ infection. Although these AMPs were not activated in our infection model, innate immunity-promoting CRAMP may work in concert with other innate or adaptive immunity-promoting AMPs in some infection systems, particularly those involving luminal-dwelling parasites. Since macrophages are generally activated at the initial infection stage,^52^ it remains to be investigated whether CRAMP can dominate the early response. These findings help to explain how a diverse AMP repertoire can be deployed in distinctive immunological settings.

Another relevant question of significant interest is the primary source of the CRAMP-producing macrophages in regulating gut microbiota. In this study, we used a peripheral infection model. Although our cell transfer experiments indicate that the peritoneal macrophage can promote the growth of *Lactobacillus* bacteria, it remains unclear whether they influence gut microbiota mainly by releasing CRAMP into the bloodstream or by migrating to the intestinal site. The observation of higher CRAMP levels or CRAMP-producing macrophages in the blood compared to colon tissue suggests the involvement of an extra-intestinal CRAMP signal in the regulation. Additionally, since the FITC dextran-labeling experiment demonstrated the migration of peritoneal macrophages into the colon lamina propria, it is possible that peritoneal macrophages also play a role in regulating gut microbiota upon their entry into the intestine. Thus, our results support the idea that CRAMPs from both serum and local macrophages having been migrated from peritoneal cavity into the intestine can influence gut bacterial communities. However, the primary source of CRAMP in this context remains uncertain. Furthermore, tissue-resident macrophages may also be activated by circulating helminth ESP proteins and contribute to this regulatory process (Figure 7).

We have shown that the dietary nutrient, vitamin D, can control the CRAMP-based remodeling of gut microbiota during helminth infection by suppressing CRAMP signaling via TLR-triggered pathways. Although this is the first report to show the influence of diet on the interaction between helminths and host gut microbiota, vitamin D is known to have numerous effects on immunity and infection, including modulation of the innate and adaptive immune responses.^53^ For example, it has been shown to promote the CRAMP against various intracellular pathogens, including *M. tuberculosis* ^43^ in humans. Our findings reveal a new way vitamin D downregulates TLR-mediated CRAMP expression and blocks the associated changes to the microbiota, thereby inhibiting immunosuppression and reducing parasite burden in a mouse model. This suggests that vitamin D could be used as a regulator in treating parasite infection or helminth-associated autoimmune-related diseases. However, this therapeutic potential remains to be investigated in humans, as our vitamin D-dependent pathway is different from that in mice.^44,54^ Nevertheless, our results support the idea that diet can influence the interplay between parasites and host microbiota. This is an important finding as current experimental or clinical helminth therapies against autoimmune and other inflammatory disorders, or treatments against parasite infections, rarely take diet or nutrients into account.^55^

Many studies have found an association between helminth infection and decreased prevalence of autoimmune or inflammatory diseases, linked to remodeling of the host microbiota.^5,6,56^ Our findings complement and extend these studies by demonstrating that CRAMP signaling bolsters immunoregulation and drives the induction of Treg cells by shifting the gut microbiota toward an immunosuppressive microenvironment. Notably, since CRAMP is involved in innate immunity,^57^ it is possible that signaling via macrophage TLRs directly regulates immunity during helminth infection. Our results indicate an association between lactobacilli expansion and Treg cell induction, which is in line with previous studies showing that lactobacilli can promote Treg cells and enhance Treg cell-mediated responses during *H. polygyrus* infection.^15,46^ However, we cannot exclude that other microbes whose abundance is changed during helminth infection also contribute to mucosal immune regulation. In addition, although we showed that helminth-derived ESP, which is the major effector for host–parasite interactions, is effective in activating the TLR pathway, the exact proteins or metabolites remain to be determined.

In summary, our results reveal a mechanism through which the antibacterial program of macrophages, triggered by ESP through TLR pathways, directs lactobacilli expansion during helminth infection, which bolsters Treg cell expansion and parasite survival. We further show that this regulation is suppressed by dietary vitamin D. This has implications for the gut microbiome-targeted or dietary nutrient-based interventions of parasitic infections and inflammatory disorders.

## Supplementary Material

Supplementary_files.doc

## Data Availability

16S rRNA gene sequencing data and RNA-seq sequencing data are available at GenBank under the accession number PRJNA2956.

## Code availability

No custom code was developed for the analysis of data in this manuscript.
