# Supplementary material for: Helminth reshapes host gut microbiota and immunoregulation by deploying an antimicrobial program of innate immunity
Source: Gut Microbes. 2025 Apr 23;17(1):2496447. doi: 10.1080/19490976.2025.2496447 (PMC12026035; doi:10.1080/19490976.2025.2496447)
Supplement: Supplementary_files.doc [file KGMI_A_2496447_SM0329.doc]

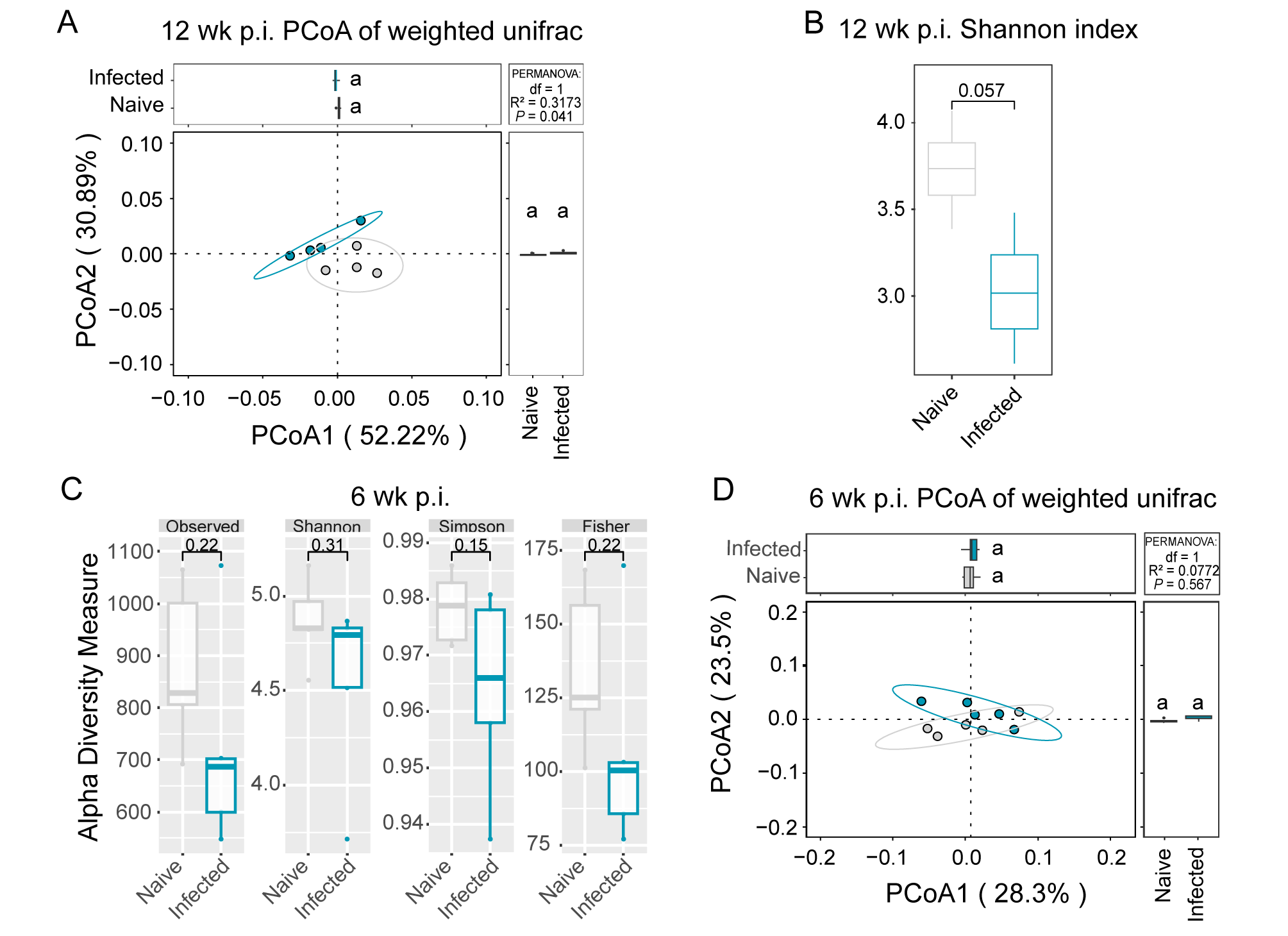


**Fig. S1.** **The gut microbiome is significantly changed in *Emu* infection.** **(A)** PCoA with 95% confidence ellipse for the gut microbiome (16S rRNA sequencing of colon content) based on weighted Unifrac distance from the mice at week 12 post-infection. **(B)** Comparison of Shannon Index between naive and *Emu* infection groups. **(C)** Comparison of alpha diversity index between naive and *Emu* infection groups after 6 weeks post-infection. **(D)** PCoA with 95% confidence ellipse for the gut microbiome (16S rRNA sequencing of colon content) based on weighted Unifrac distance for the mice at 6 weeks post-infection. n = 4 mice per group for panels A and B or n = 5 mice per group for panels C and D.The box plot represents the 25th percentile, median, and 75th percentile and whiskers stretch to 1.5 times the interquartile range from the corresponding hinge. *P* values were determined by the PERMANOVA test for panels A and D or two-sided Wilcoxon rank-sum test for panels B and C.


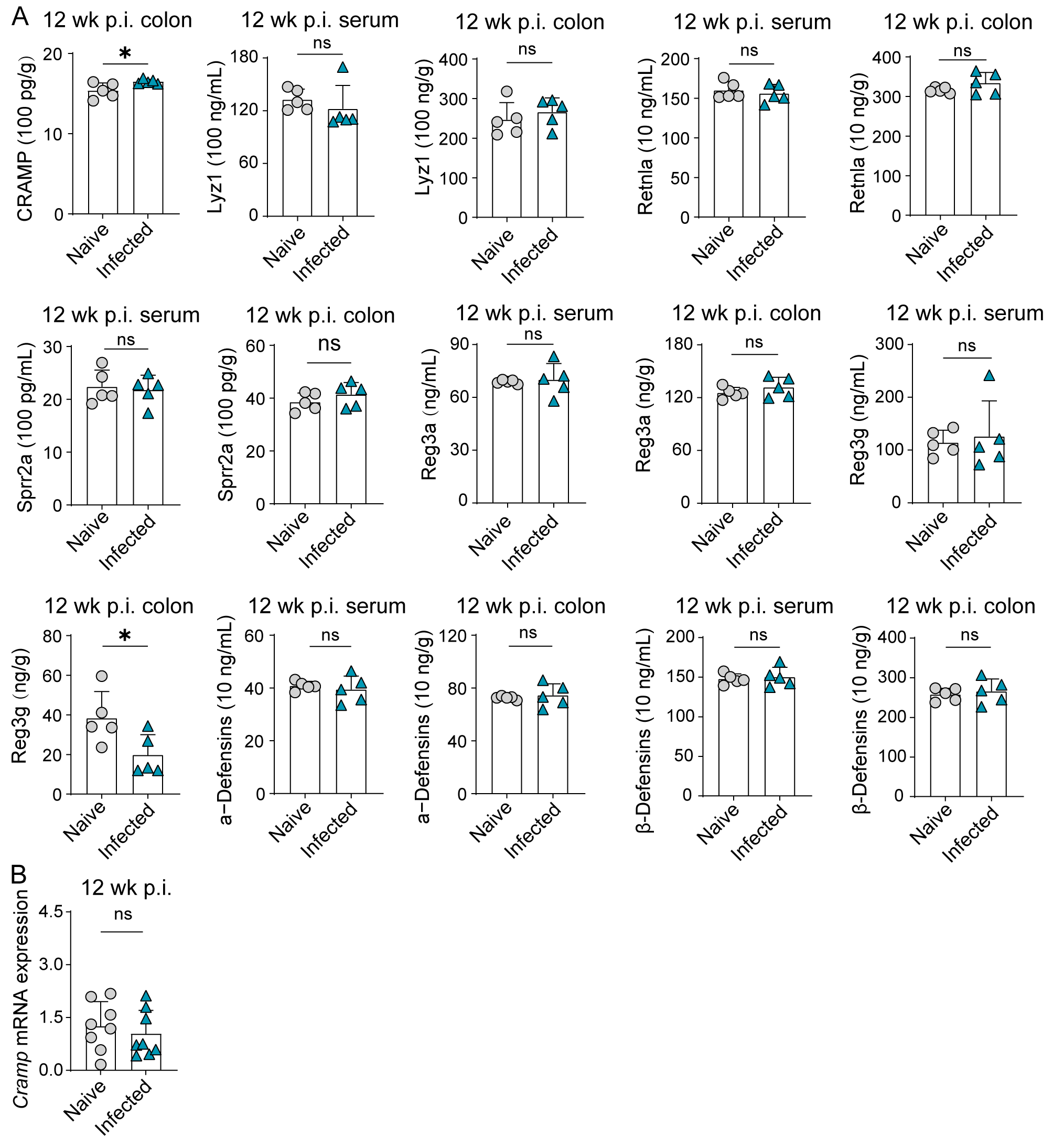


**Fig. S2. ELISA measurements of antibacterial peptides (AMPs) in serum and colon tissue in infection.** **(A)** The protein level of AMPs in the serum and colon tissue of the mice at week 12 post-infection. **(B)** qPCR analysis of the expression level of *Cramp* in the intestinal epithelial cells isolated from the mice at 12 weeks post-infection. Data presented as mean ± s.d, with mouse numbers indicated in each panel. n = 5 mice per group for panel A or n = 8 mice per group for panel B. The *P* values were determined by the two-sided Student's t-test for panels A and B (**P* < 0.05).


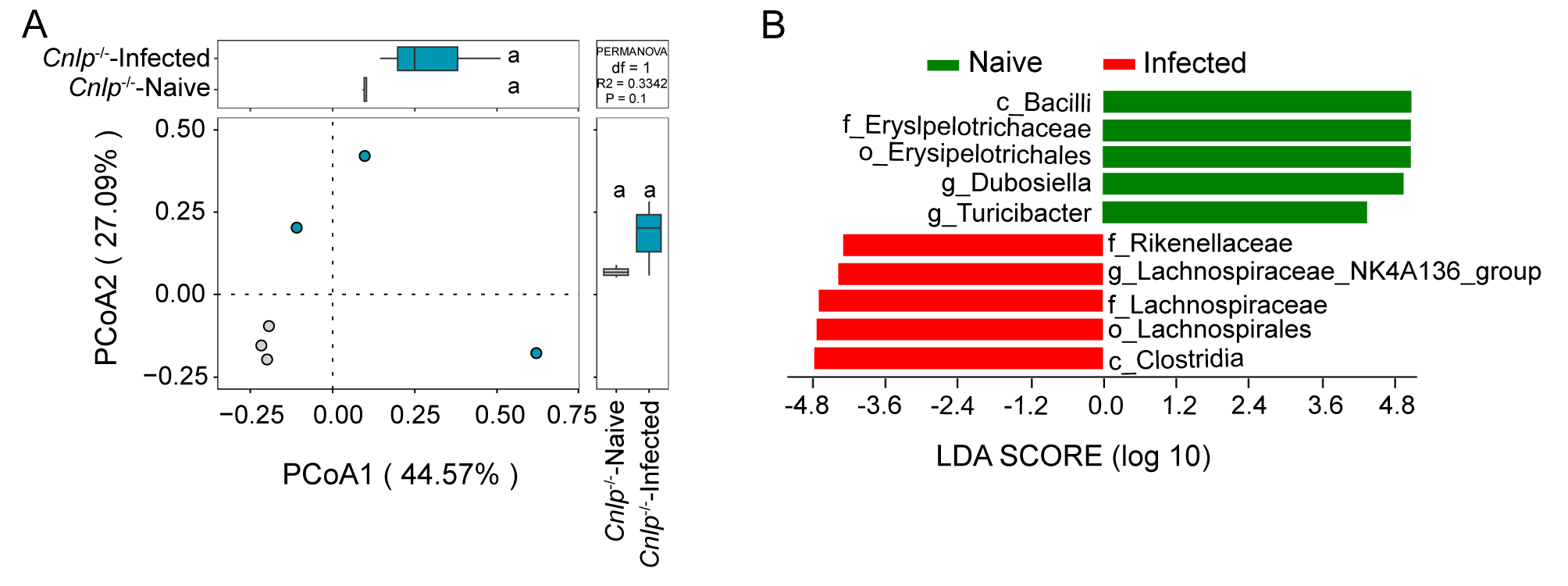


**Fig. S3. The gut microbiome changes in the infection of *Cnlp^-/-^* mice.** **(A)** PCoA for the gut microbiome (16S rRNA sequencing of colon content) based on Bray-Curtis distance from the *Clnp^-/-^* mice at 12 weeks post-infection. **(B)** Linear discriminant analysis Effect Size (LEfSe) analysis between the gut microbiome for naive and infected *Clnp^-/-^* mice at 12 weeks post-infection. Only the taxa with LDA score (log 10) > 4 are shown. n = 3 mice per group. The *P* value was determined by the PERMANOVA test for panel A.


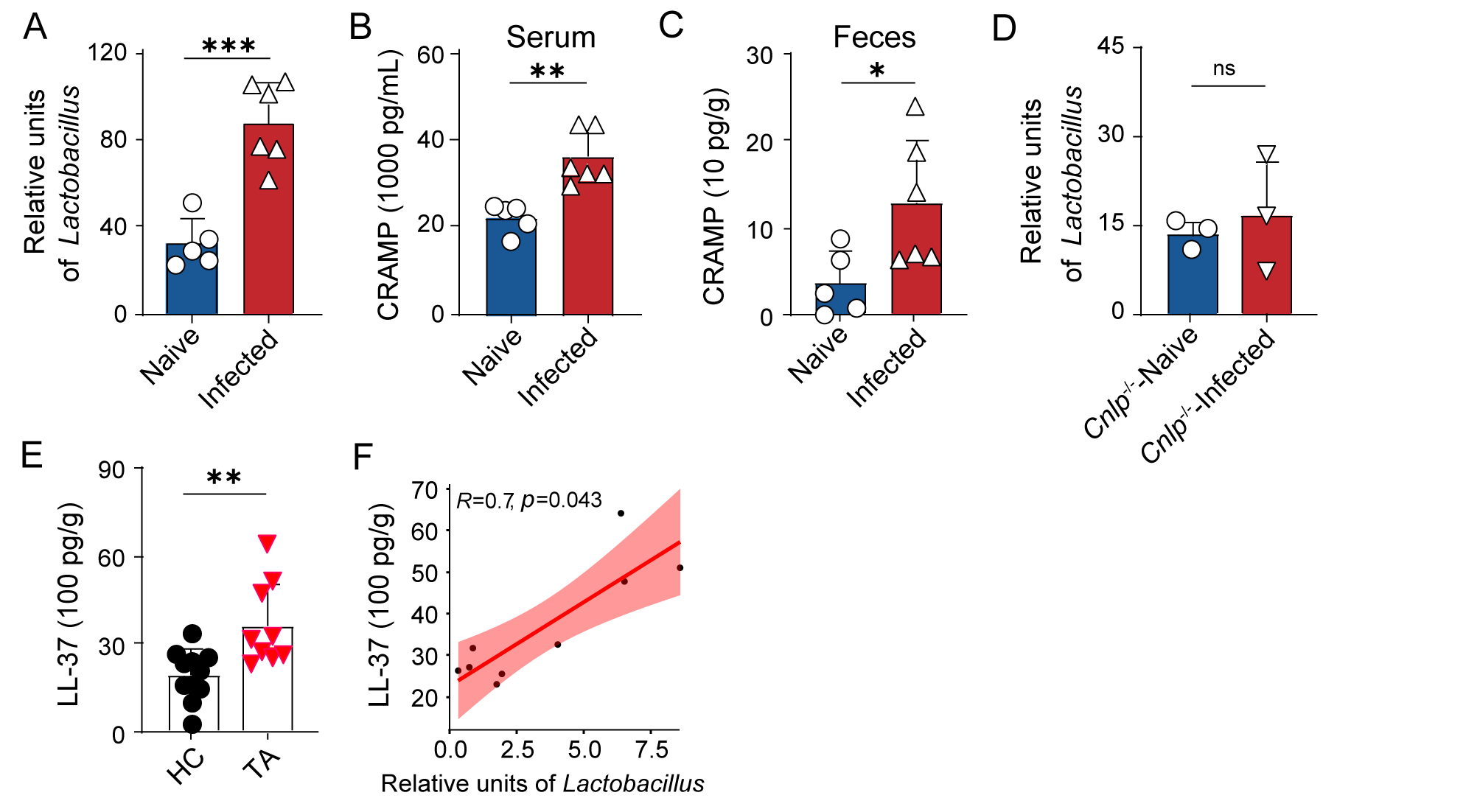


**Fig. S4. CRAMP correlates the expansion of lactobacilli bacteria during other helminth infections.** **(A)** qPCR analysis of *Lactobacillus* abundance in feces of naive mice and infected mice at 8 weeks post-infection of *Trichinella spiralis*. **(B)** ELISA measurement of CRAMP in the serum of the mice at 8 weeks post-infection of *T. spiralis*. **(C)** ELISA measurement of CRAMP in the feces of the mice at 8 weeks post-infection of *T. spiralis*. **(D)** qPCR analysis of *Lactobacillus* in feces of the naive mice and infected *Clnp^-/-^*mice at 8 weeks post-infection of *T. spiralis*. **(E)** ELISA analysis of LL-37 in the feces of the health control (HC) and *Taenia asiatica* infected (TA) individuals. **(F)** Spearman correlation between the protein level of LL-37 and the abundance of *Lactobacillus* in the feces of *T. asiatica*-infected individuals. Data presented as mean ± s.d for panels A-E. Mouse numbers are indicated in each panel. The *P* values were determined by the two-sided Student's t-test for panels A-E or by Spearman's rank correlation coefficient test for panel F (**P* < 0.05, ***P* < 0.01, ****P* < 0.001).

**
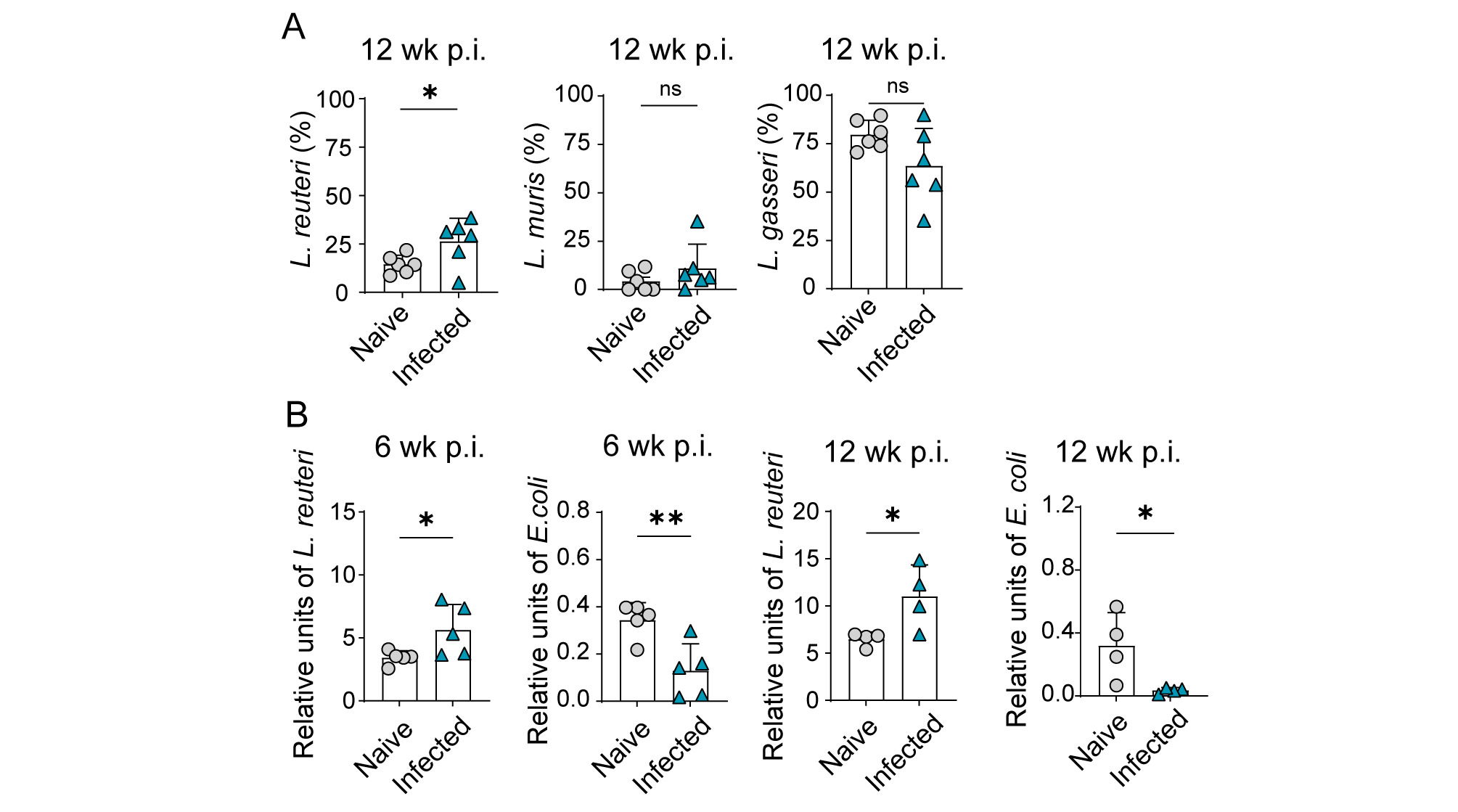
**

**Fig. S5. Colony identification of *Lactobacillus* species in the feces in infection.** **(A)** Colony frequency of the *L. reuteri*, *L. muris*, and *L. gasseri* isolates in the feces of the mice. The ratio represents the proportion of each isolate in the total colonies identified from the feces of mice at 12 weeks post-infection. **(B)** qPCR analysis of the abundances of *L. reuteri* and *E. coli* in the feces of the naive or infected mice at different infection stages. Data presented as mean ± s.d. Results are representative of two independent experiments with 4-6 mice per group. The *P* values were determined by the two-sided Student's t-test for panels A and B (**P* < 0.05, ***P*< 0.01).


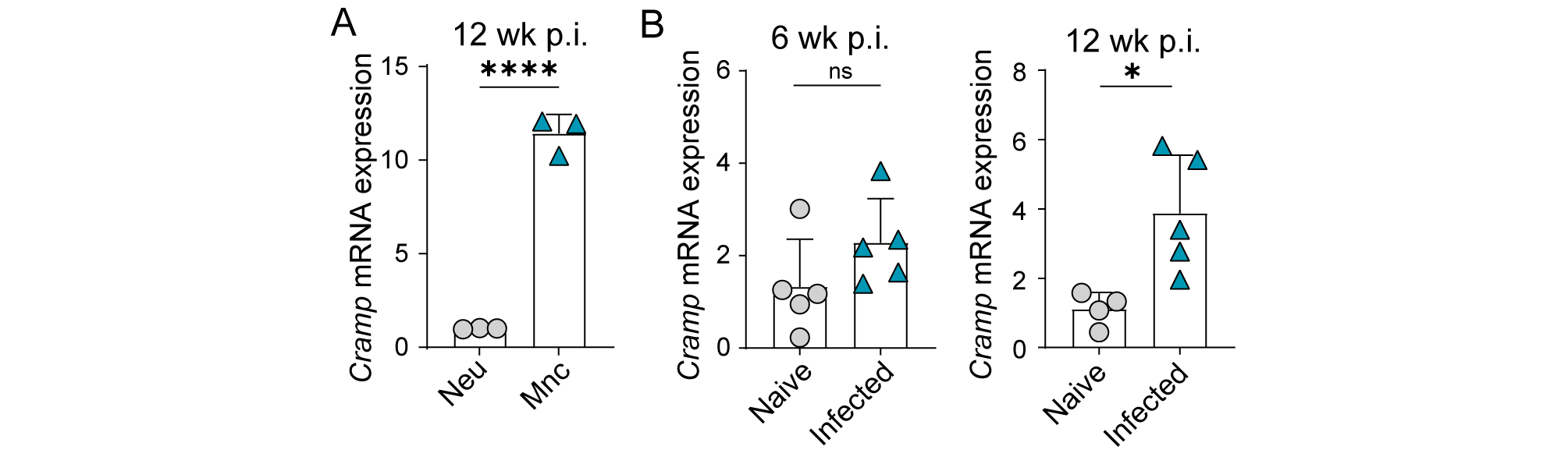


**Fig. S6. Analysis of the main sources of CRAMP in infection.** **(A)** qPCR analysis of the expression level of *Cramp* in the granulocytes (Neu) and mononuclear cells (Mnc) in the peripheral blood of the mice at 12 weeks post-infection. **(B)** qPCR analysis of the expression level of *Cramp* in the peritoneal macrophages isolated from the mice at 6 weeks (left) or 12 weeks post-infection (right). Data presented as mean ± s.d. Results are representative of two independent experiments with mice numbers shown in each panel. The *P* values were determined by the two-sided Student's t-test for panels A and B (**P* < 0.05, *****P* < 0.0001).


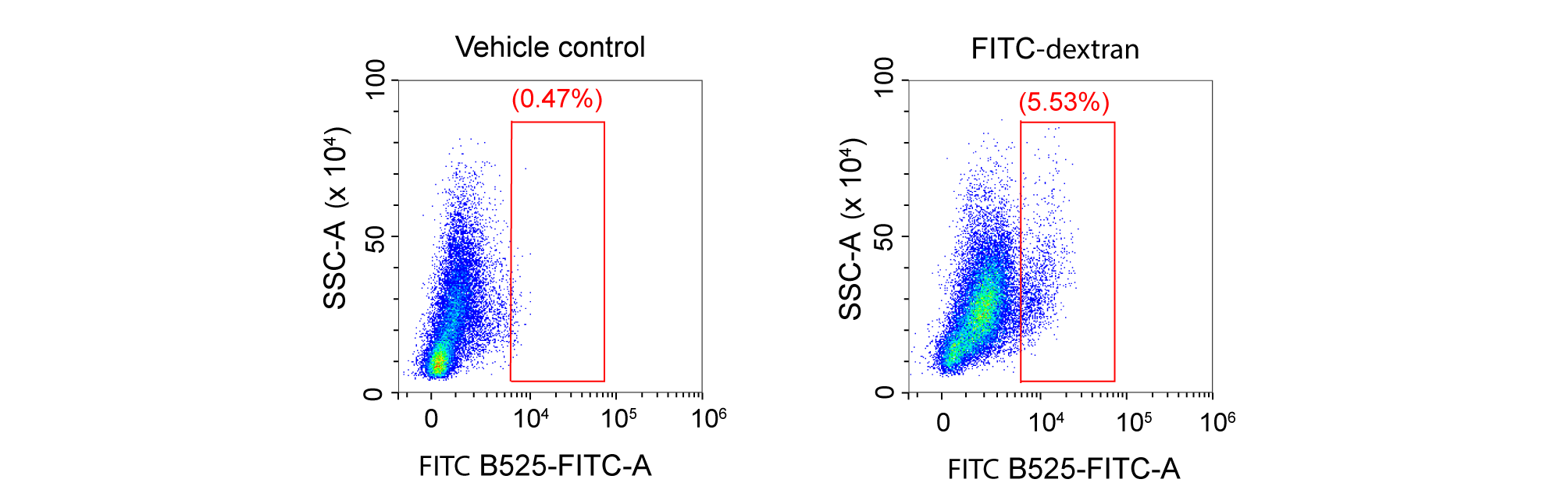


**Fig. S7. The flow cytometry analysis of peritoneal macrophages with FITC-dextran.** Peritoneal macrophages were isolated, labeled with vehicle control (left) or FITC-dextran (right), and injected into the mouse peritoneal cavity. The FITC-dextran fluorescence of macrophages at the colon lamina propria was analyzed using flow cytometry.


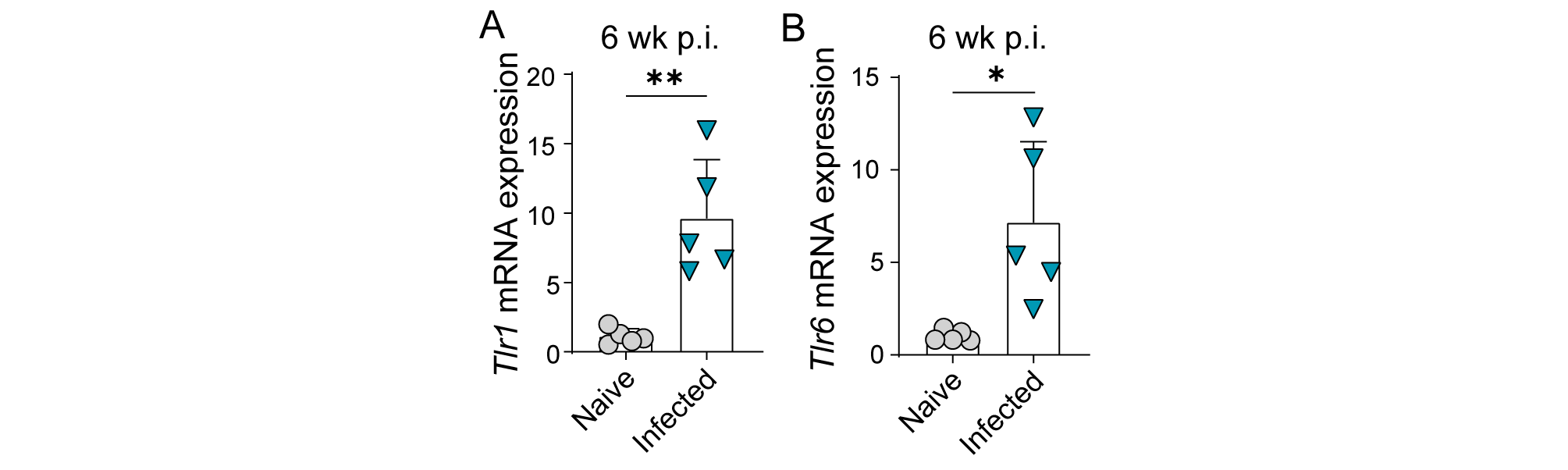


**Fig. S8. The expression levels of *Tlr1* and *Tlr6* in the peritoneal macrophages in infection. (A)** qPCR analysis of the expression level of Tlr1 in the peritoneal macrophages isolated from the naive mice and infected mice at 6 weeks post-infection. **(B)** qPCR analysis of the expression level of *Tlr6* in the peritoneal macrophages isolated from the naive mice and infected mice at 6 weeks post-infection. Data presented as mean ± s.d. Results are representative of two independent experiments with 5 mice per group. *P* values were determined by the two-sided Student's t-test for panels A and B (**P* < 0.05, ***P* < 0.01).


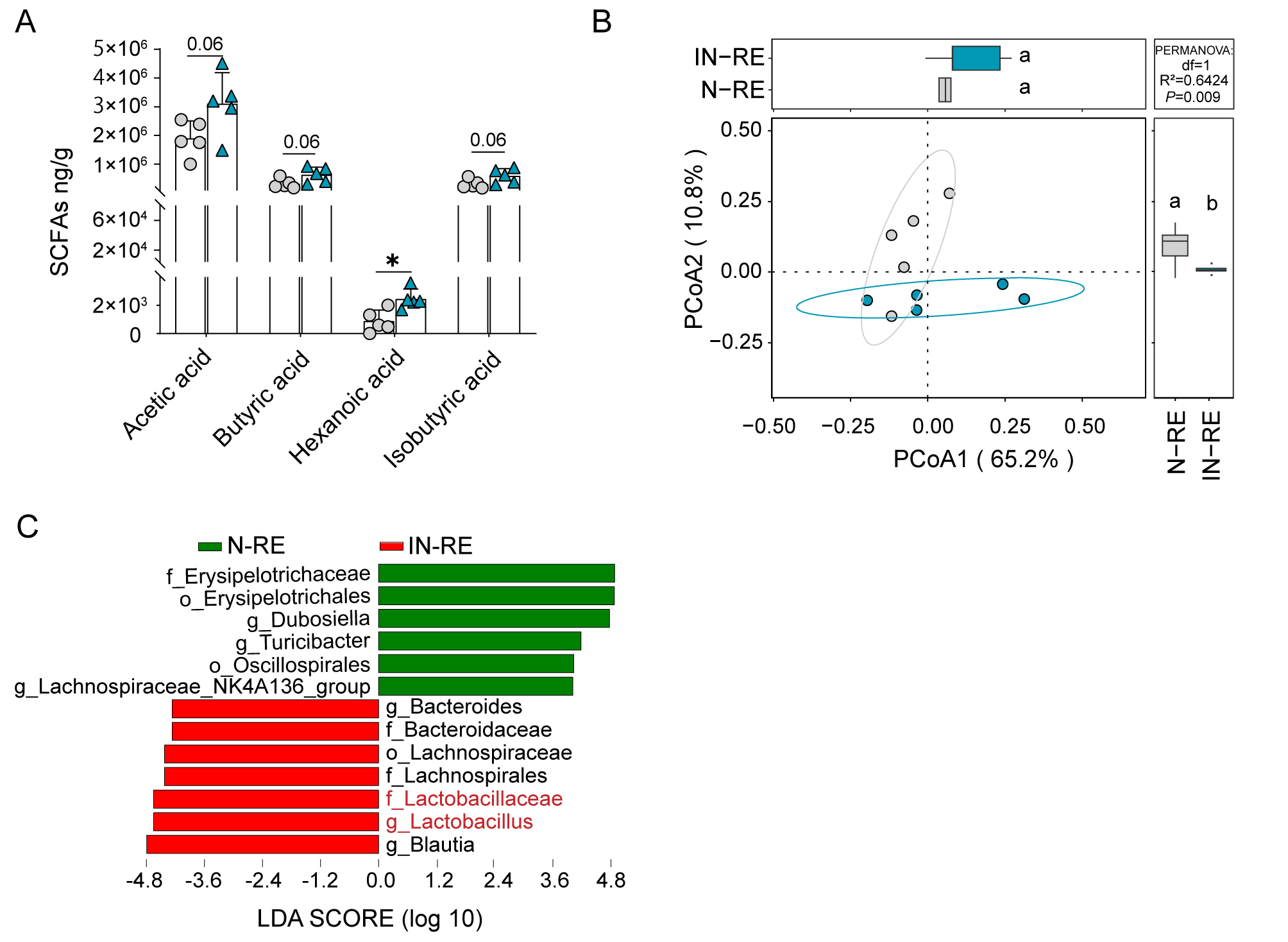


**Fig. S9. Metabolomic and metagenomic features in the mice in fecal microbiota transplantation (FMT).** **(A)** Targeted metabolomic analysis of Short-chain fatty acids (SCFAs) in infection of donor mice. **(B)** PCoA based on Bray-Curtis distance with 95% confidence ellipse for the gut microbiome(16S rRNA sequencing of colon content) of the recipient mice after FMT. The recipient mice (RE) were pretreated with antibiotics (ABX) cocktails for 5 days, followed by an FMT regime of 1 week from naive donors (N) or Emu-infected donors (IN) at week 12 post-infection. **(C)** Linear discriminant analysis Effect Size (LEfSe) analysis for the gut microbiome of recipient mice. Only the taxa with LDA score (log 10) > 4 are shown. The mice numbers are n = 5 per group. P values were determined by the two-sided Student's t-test for panel A or by the PERMANOVA test for panel B (**P* < 0.05).

**
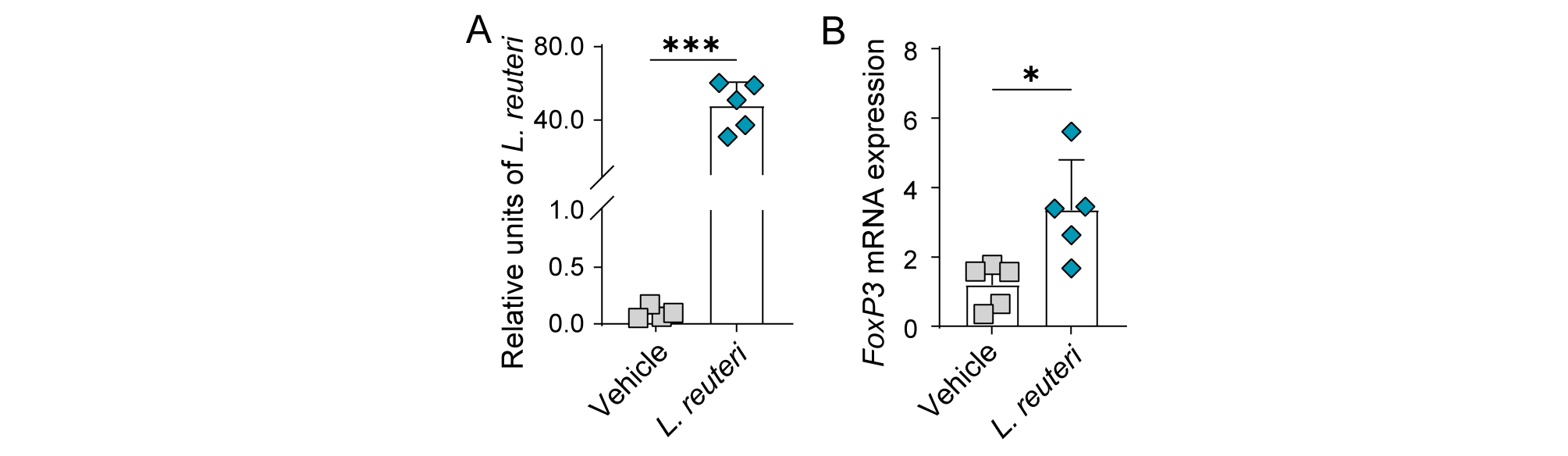
**

**Fig. S10. The *L. reuteri* isolate promotes colonic *Foxp3* expression.** **(A)** qPCR analysis of the abundances of *L. reuteri* in the colonic contents of mice. The mice were orally gavaged with 2 × 10^9^ CFUs of *L. reuteri* isolate in 200 μL PBS or PBS control daily for 2 weeks. **(B)** qPCR analysis of the expression level of *Foxp3* in the colon tissue. Data presented as mean ± s.d. Results are representative of two independent experiments with mice numbers shown in each panel. The mouse numbers are shown in each panel. *P* values were determined by the two-sided Student's t-test (**P* < 0.05, ****P* < 0.001).


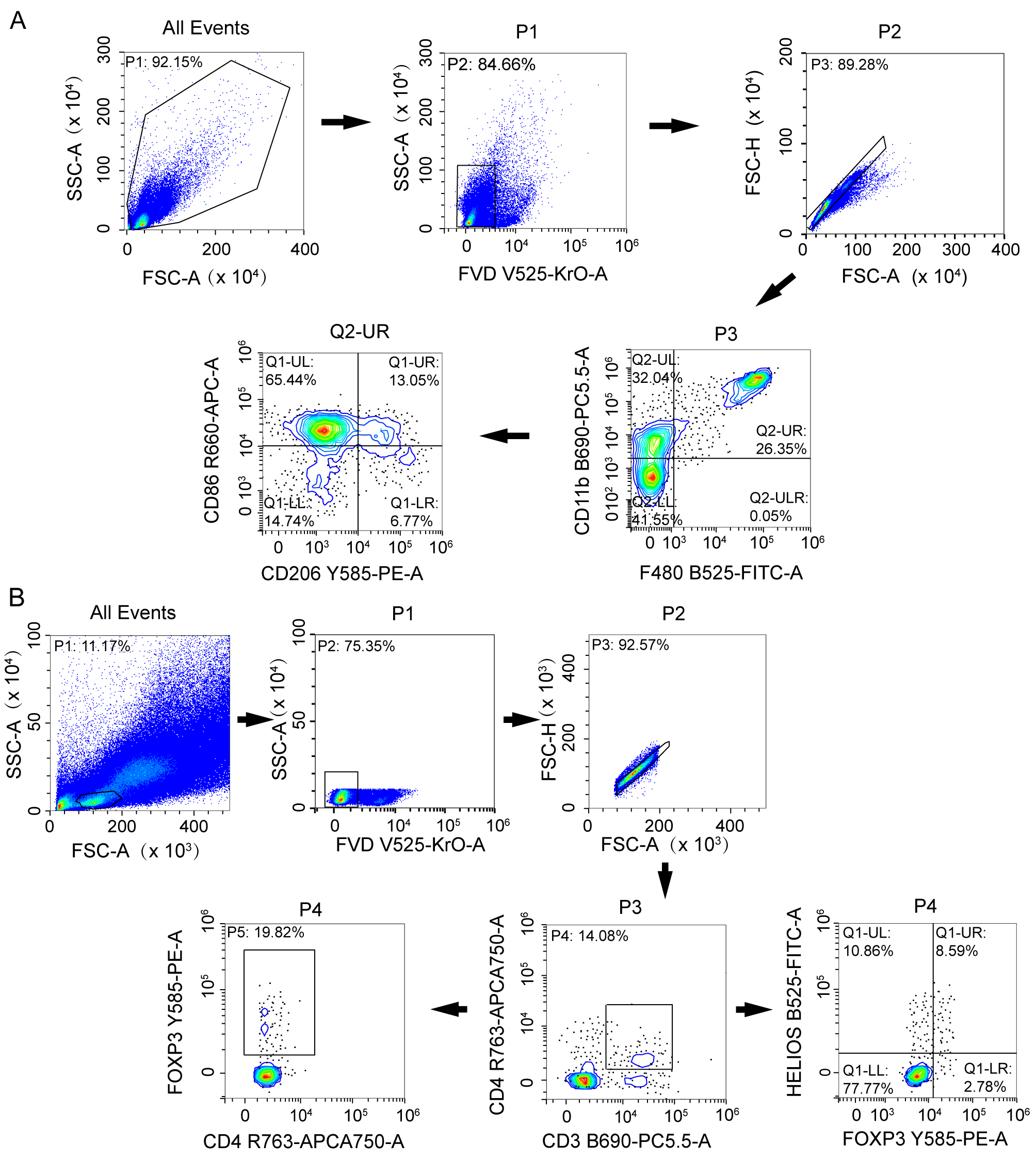


**Fig. S11. The gating strategies for flow cytometry analyses.** **(A)** The flow cytometry gating strategy for peritoneal macrophages. First, monocytes were gated, followed by the removal of adherent cells and identification of viable, FVD-negative cells. Macrophages were then selected based on F4/80^+^CD11b^+^ expression, and the CD86^+^CD206^-^ and CD86^-^CD206^+^ subsets were identified. **(B)** The flow cytometry gating strategy for colonic lymphocytes. First, forward and side scatter gating was applied to exclude debris and adherent cells. Viable cells were identified by excluding FVD-positive cells. CD4^+^ T helper cells were identified as CD3^+^CD4^+^ subset. Treg cell populations were further characterized from CD4^+^ T cells by the expression of CD4^+^Foxp3^+^ and CD4^+^Helios^-^Foxp3^+^.

Table S1. The qPCR or sequencing primers in this study.

| **Gene locus** | **Primer name** | **Sequence (5'-3')** |
| --- | --- | --- |
| *Lactobacillus* | LabF362 | 5'-AGCAGTAGGGAATCTTCCA-3' |
|  | LabR677 | 5'-CACCGCTACACATGGAG-3 |
| Enterobacteriaceae | Uni515F | 5'-GTGCCAGCAGCCGCGGTAA-3' |
|  | Ent826R | 5'-GCCTCAAGGGCACAACCTCCAAG-3' |
| *Lactobacillus reuteri* | F1 | 5'-CAGACAATCTTTGATTGTTTAG-3' |
|  | R1 | 5'-GCTTGTTGGTTTGGGCTCTTC-3' |
| *Escherichia coli* | F1 | 5'-CATGCCGCGTGTATGAAGAA-3' |
|  | R1 | 5'-CGGGTAACGTCAATGAGCAAA-3' |
| 16S V3-V4 | 341-F | 5'-CCTAYGGGRBGCASCAG-3' |
|  | 806R | 5'-GGACTACHVGGGTWTCTAAT-3' |
| 16S V4 | 515F | 5'-GTGCCAGCMGCCGCGGTAA-3' |
|  | 806R | 5'-GGACTACHVGGGTWTCTAAT-3' |
| *Cramp* | F1 | 5'-GGCTGTGGCGGTCACTAT-3' |
|  | R1 | 5'-GTCTAGGGACTGCTGGTTGAA-3' |
| *Foxp3* | F1 | 5'-ACCATTGGTTTACTCGCATGT-3' |
|  | R1 | 5'-TCCACTCGCACAAAGCACTT-3' |
| *IL-10* | F1 | 5'-TTCTTTCAAACAAAGGACCAGC-3' |
|  | R1 | 5'-GCAACCCAAGTAACCCTTAAAG-3' |
| *Tlr1* | F1 | 5'-GGTGTTAGGAGATGCTTATGGGG-3' |
|  | R1 | 5'-GATGTTAGACAGTTCCAAACCGA-3' |
| *Tlr2* | F1 | 5'-TCTGGGCAGTCTTGAACATTT-3' |
|  | R1 | 5'-AGAGTCAGGTGATGGATGTCG-3' |
| *Tlr6* | F1 | 5'-TTTGCGCCCTGGCCTTAATA-3' |
|  | R1 | 5'-GCAGGTCTTTGGGGACATGA-3' |
| *Vdr* | F1 | 5'-CACCTGGCTGATCTTGTCAGT-3' |
|  | R1 | 5'-CTGGTCATCAGAGGTGAGGTC-3' |
| *Cyp27b1* | F1 | 5'-CCGCGGGCTATGCTGGAAC-3' |
|  | R1 | 5'-CTCTGGGCAAAGGCAAACATCTGA-3' |
| *Rps13* | F1 | 5'-GTCCGAAAGCACCTTGAGAG-3' |
|  | R1 | 5'-AGCAGAGGCTGTGGATGACT-3' |
| *B2M* | F1 | 5'-TTCTGGTGCTTGTCTCACTGA-3' |
|  | R1 | 5'-CAGTATGTTCGGCTTCCCATTC-3' |
| *GAPDH* | F1 | 5'-CATCACTGCCACCCAGAAGACTG-3' |
|  | R1 | 5'-ATGCCAGTGAGCTTCCCGTTCAG-3' |
